# Ecological study of the association between the English national vaccination programme and area deprivation inequalities in COVID-19 mortality

**DOI:** 10.1136/bmjopen-2024-085195

**Published:** 2025-01-21

**Authors:** Natalie Bennett, Clare Bambra, David Sinclair, Adam Todd, Fiona Matthews

**Affiliations:** 1 Population Health Sciences, Newcastle University, Newcastle upon Tyne, UK; 2 School of Pharmacy, Newcastle University, Newcastle upon Tyne, UK; 3 Institute for Clinical and Applied Health Research, University of Hull, Hull, UK

**Keywords:** COVID-19, Mortality, Vaccination, PUBLIC HEALTH

## Abstract

**Abstract:**

**Objective:**

To understand how area deprivation inequalities in COVID-19 mortality changed during the national vaccination programme in England and to identify the extent to which these inequalities might be explained by unequal vaccination uptake.

**Design:**

Ecological study.

**Setting:**

307 Lower Tier Local Authorities in England, March 2020 – December 2022.

**Main outcome measure:**

Inequality in age-standardised mortality rates 28 days after a positive COVID-19 test by area-level deprivation from March 2020 to December 2022. We employ three different measures of this inequality: the disparity index, the concentration and generalised concentration index, and absolute and relative measures of inequality. We use the 2019 edition of the Index of Multiple Deprivation, transformed into quintiles.

**Results:**

Relative inequalities in age-standardised mortality rates 28 days after a positive COVID-19 test reduced substantially (from around 6.9 times higher in most deprived to least deprived to 1.2 times higher) in the 25 months after the national vaccination rollout began. Vaccination uptake between the most and least deprived quintiles widened with each dose. Inequalities in cumulative mortality rates developed quickly, and while they stabilised and reduced, they did not disappear. We estimate that if vaccination rates in the most deprived areas had been the same as those in the least deprived, absolute disparity inequality would have been reduced from 118.9 per 100 000 (95% CI 117.0 to 120.7) to 40.2 (95% CI 3.7 to 76.7) at the end of 2022.

**Conclusions:**

National COVID-19 vaccination strategies offer the potential to significantly reduce inequalities in COVID-19 mortality rates. However, more could be achieved if barriers to vaccination uptake in the most deprived areas are overcome.

**STRENGTHS AND LIMITATIONS OF THIS STUDY**

- We assessed population-wide inequalities via area-level deprivation.
- This enabled us to model the difference between observed inequalities in vaccination rates and hypothetical inequalities if uptake in the most deprived areas had been more similar to the least deprived.
- This is an ecological study and results pertain to areas, not individuals.
- We were unable to disentangle the effects of the periods of ‘lockdown’ as they were applied equally to the whole country.

## Introduction

 COVID-19 has been associated with just over 7 million deaths globally.^1^ Despite initial claims that the virus ‘does not discriminate’, evidence suggests that, in many countries, COVID-19 mortality has been unequal—higher—in lower socioeconomic groups and in more deprived areas.^2^ This has also been observed in a UK context, with substantial inequalities by area deprivation.^3^ Explanations for this inequality include unequal vulnerability, susceptibility, exposure and transmission.^4^ These pathways include a higher prevalence of comorbidities and poverty, as well as a greater proportion of the population working in the service sector (or ‘essential worker’ roles), and living in densely populated areas with household overcrowding.^4^ COVID-19 has since been called an ‘unequal pandemic’, with far reaching consequences for health and inequalities.^5^ Concerns surrounding the impact of our response to the pandemic in terms of the potential to further exacerbate these inequalities were also raised in the early stages of the pandemic.^6^

Population-wide vaccination is now a core containment strategy employed worldwide. Globally, estimates suggest that the first year of the COVID-19 vaccination effort (2021) prevented 19.8 million deaths.^7^ The UK was the first country to approve a COVID-19 vaccine.^8^ Vaccination was rolled out according to clinical vulnerability and age.^9^ By July 2021, a first dose had been offered to everyone over 18 years in the UK.^10^ The full vaccine schedule available to every adult in the UK includes two initial doses, along with a first booster dose, made available to all adults in response to the Omicron variant in 2021.^11^ Subsequent booster doses were made available to those over 50 years (increased to over 65 years only in 2023), those aged between 5 and 49 years who are clinically at risk, and those working in care or front-line health services.^12^ Evidence suggests that the full course of vaccination, with the addition of the first booster (three doses in total), offers high levels of antibodies to those who receive it.^13^ Overall, compared with other high-income countries, initial vaccination uptake in the UK was high.^14^ Despite this initial success, inequalities in uptake by area-level deprivation emerged as the vaccination programme was expanded across the whole adult population.^15^ It is not yet clear what impact this inequality in vaccination uptake may have on inequalities in COVID-19 mortality. As vaccination remains a key policy in the transition towards ‘living with’ COVID-19 for many countries, understanding how vaccination inequalities impact mortality inequalities is of enduring importance.

A small number of studies have examined the association between vaccination and inequalities in COVID-19–related mortality to date. These studies largely only analyse the overall proportion of the population vaccinated and its relationship with COVID-19 mortality inequality. A study in England which analysed early data (deaths up to April 2021) demonstrated a weakening area-level association between socioeconomic factors and COVID-19 deaths during the first 4 months of the vaccine rollout.^16^ Another study using data covering a year of the vaccination rollout (up to December 2021) in Bavaria, Germany, described an observed reduction in inequalities in COVID-19 mortality by area deprivation during the period of vaccination.^17^ Similarly, a US-based study, using county-level data, reported that the association between area-level socioeconomic status and the COVID-19 case fatality rate was mediated by vaccination coverage.^18^ Finally, a more recent global study of 161 countries and 58 states also demonstrated that, compared with a hypothetical scenario of no vaccination, changing from a scenario of the greatest disparity in vaccination rates to any other less unequal scenario was associated with a large proportion of deaths which could have been averted.^19^ Research explicitly analysing COVID-19 vaccination inequality and its relationship with COVID-19 mortality inequality has not yet been conducted.

In this study, we aim to understand how inequalities by area-level deprivation in COVID-19 mortality changed during the rollout of the national vaccination strategy (December 2020 to December 2022) in England and to identify the extent to which these inequalities are associated with unequal vaccination uptake. This study therefore aims to determine whether relative and absolute inequality in COVID-19 mortality decreased in association with the COVID-19 vaccination programme in England.

## Methods

### Study design and data sources

In this ecological analysis, we use publicly available data^20^ on deaths 28 days after a positive COVID-19 test and vaccination uptake in England and link this to area-level deprivation data. All analyses were performed using Stata 18. We employ two primary measures: vaccination doses and deaths within 28 days of a positive COVID-19 test data. These data are available daily. It was not possible to disaggregate our analysis further as sex stratified mortality data were not available. We operationalise the data at the Lower Tier Local Authority (LTLA) scale. LTLAs are administrative bodies which are responsible for the provision and maintenance of public services in a defined area. There are 309 LTLAs in England, 307 of which were used in our analysis due to exclusion of the two smallest LTLAs due to censored data: the City of London and the Isles of Scilly. The size of LTLA populations ranges from 41 000 (Rutland) to 1 158 000 (Birmingham) with a mean of 194 000 persons (SD 130 000, SE 7600) and a median of 149 000 persons. LTLAs are subcomponents of regions. We employ the LTLA scale in order that meaningful geographic inequalities can be estimated, while balancing data availability and sufficient mortality data. Further information on data availability and scale constraints is provided in online supplemental file 1

#### Cumulative vaccinations

We use data on vaccination uptake at the LTLA level for all three of the primary vaccination doses (first, second and first booster) for the period from the week commencing 7 December 2020 until the week commencing 26 December 2022. Using 2021 LTLA population estimates,^21^ we produced a cumulative proportion vaccinated measure for each of the three doses. An overall estimate for the proportion of the population vaccinated was created using factor analysis of the proportion who have one, two or three vaccines.^22^ Factor loadings are shown inonline supplemental file 1. We do this so that the complex process of vaccine rollout is combined so that a measure of the total population vaccinated at each time point is included in the model while balancing model parsimony. We refer to this measure as the population vaccination load.

#### Death within 28 days of a positive COVID-19 test

In this study, our outcome of interest is death within 28 days of a positive COVID-19 test (lab-based PCR tests), by date of death, registered to the person’s usual area of residence. It should be noted that deaths are counted in this measure regardless of whether COVID-19 was ultimately listed as a cause of death on the death certificate. The implications of this are reflected on in the discussion. Age-standardised deaths are not produced at the LTLA level due to identification risks. We therefore undertook a multistep process to calculate age-standardised mortality rates 28 days after a positive COVID-19 test from data on regional (a larger geography than LTLAs) age-stratified deaths (by 5 year age bands), LTLA total deaths and LTLA population age structure (by 5 year age bands). There are nine regions in England which LTLAs are nested within. In estimating the mortality data in this way, we assume a uniform distribution of mortality rates by age across differing levels of area deprivation within each region. A comprehensive definition of the outcome measure and process for calculating age-standardised mortality rates at the LTLA level, from regional data, is described inonline supplemental file 1. Further information on changes to access to COVID-19 testing for the general public and discussion surrounding the potential implications of this is also provided inonline supplemental file 1.

#### Deprivation

We used England’s official measure of deprivation, the English Index of Multiple Deprivation (IMD) from 2019, to calculate LTLA-level deprivation (see online supplemental file 1). It produces a ranking of areas in England based on relative local scores for income, employment, health, education, crime, access to services and living environment.

### Statistical analysis

Estimates of inequalities in age-standardised mortality rates 28 days after a positive COVID-19 test were graphically depicted using three measures of inequality: (1) the disparity index (a ratio variable calculated by dividing the mortality estimates for the most deprived quintile by the least deprived quintile)^23^ and absolute disparity measured by the difference in mortality estimates for the least to most deprived quintile, (2) the concentration and generalised concentration index of inequalities (capturing inequalities across all five deprivation quintiles)^24^ and finally, (3) absolute and relative measures of inequality (regressed on weekly values assuming a linear trend across the deprivation quintiles).^25^ Further detail on their creation is provided inonline supplemental file 1, along with a table describing each of the measures. The population vaccination load was estimated using factor loadings for the proportion who have one, two or three vaccines over time.^22^ Additional information on the factor analysis undertaken is provided inonline supplemental file 1.

Longitudinal analysis of panel data was undertaken on the log absolute and relative inequalities in age-standardised mortality rates 28 days after a positive COVID-19 test. The model included random effects for region. Using the Hausmann test, the effects for random effects and fixed effects were similar for absolute inequality (p=0.12) but not for relative inequality (p=0.002). Random effects analysis was undertaken for both outcomes for consistency. Generalised log-linear models were used to investigate absolute and relative inequality in age-standardised mortality rates 28 days after a positive COVID-19 test and its relationship with the proportion of the population vaccinated and lockdowns, with a lag for the previous weeks mean age-standardised COVID rates, adjusting for all three lockdowns. To investigate the role of vaccination, the mean proportion vaccinated differential between the most and least deprived for each region is modelled on absolute and relative inequality in age-standardised mortality rates 28 days after a positive COVID-19 test. To investigate absolute and relative inequality where there were no differences between the vaccination rates of most versus least deprived, the estimated vaccine effect was combined with the weekly differences in vaccine factor to provide a potential inequality reduction. In calculating this counterfactual, we take the within region average population vaccine coverage in the least deprived areas and use that estimate and the relationship between population vaccine coverage and mortality estimated within the region to show what would have happened in the most deprived areas had vaccine coverage been better.

To reduce issues of endogeneity (where the mortality rate and therefore the amount of inequality detectable and vaccination rates all vary over time), the mean absolute and relative inequalities (logged) in age-standardised mortality rates 28 days after a positive COVID-19 test in each region were regressed on mean vaccine uptake.^26^ As lockdowns were undertaken uniformly across all areas, a generalised mixed log-linear model with random effects for region on the mean age-standardised weekly log absolute and relative inequalities over time was used to investigate the association between lockdowns and inequality in age-standardised mortality rates 28 days after a positive COVID-19 test. Random effects for regions allowed the model to account for variation in outcomes at the regional level, for example, due to differences in population clustering, movement patterns and adoption of social distancing behaviours. Confidence intervals were calculated using 500 bootstrap samples and indicate uncertainties on the estimates of inequalities within each of the nine regions.

### Patient and public involvement

We consulted with the Health Foundation’s Inclusion Panel (https://www.health.org.uk/about-the-health-foundation/inclusion-panel). Public and practice members discussed their experiences of area-level inequalities in COVID-19 with us, influencing the conceptualisation and design of this study.

## Results

**Figure 1:**
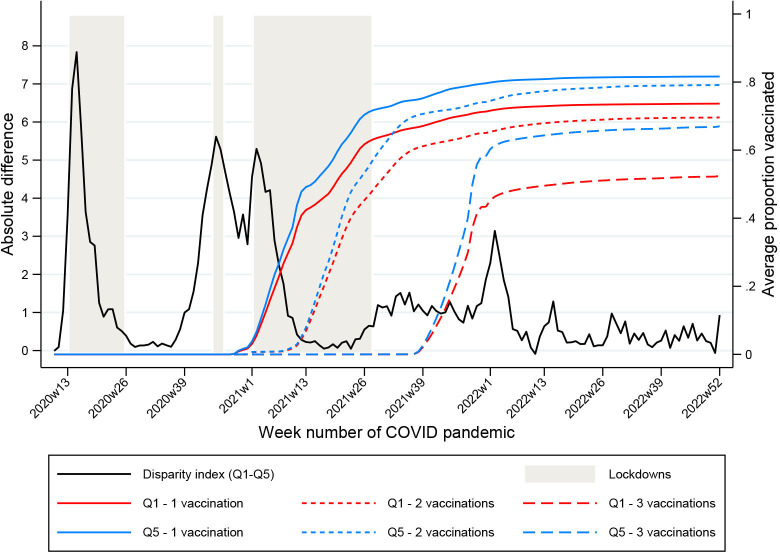
Line graph showing the absolute disparity inequality in cumulative age standardised mortality rate 28 days after a positive COVID-19 test (per 100 000) between the most (quintile one) and least (quintile five) deprived areas and the average proportion of the population vaccinated over time by dose.

Trends in vaccination uptake for the most and least deprived quintiles are shown in figure 1. This figure demonstrates that the average proportion of the population in LTLAs in the most and least deprived quintiles receiving the vaccine has declined with each dose. The average proportion vaccinated at the end of 2022 for vaccination 1 was 73.7% (SD 4.5) in the most deprived quintile and 81.6% (SD 2.3) in the least (a difference of 7.9%); for vaccination 2, it was 69.6% (SD 4.8) in the most deprived and 79.1% (SD 2.4) in the least deprived (a difference of 9.5%) and for vaccination 3, it was 52.4% (SD 7.4) in the most deprived and 67.0% (SD 3.4) in the least (a difference of 14.6%). The gap in uptake between the most and least deprived areas is wider with each vaccine dose.

Figure 1 also shows trends in cumulative absolute disparity inequalities in age-standardised mortality rates 28 days after a positive COVID-19 test. This figure shows that inequalities in cumulative mortality and vaccine uptake by deprivation persisted throughout the entire period of observation. It highlights plateaus in inequality during and immediately following the two larger periods of national lockdown and with increasing population vaccination uptake.

The first three figures in theonline supplemental file 1 show the same figure with weekly relative disparity, cumulative relative disparity and weekly absolute disparity, respectively. Relative weekly disparity inequalities were highest during the two peak mortality periods (from 5 October to 11 October 2020 and from 28 June to 4 July 2021). After the end of the third lockdown, we see a gradual decrease in relative inequality in mortality. Throughout 2022, we consistently see low levels of absolute disparity inequality in weekly mortality. During this time, there were no legally enforced social-distancing measures (such as lockdown) in place, though mask wearing and self-isolation on testing positive were recommended.

online supplemental file 1 also shows the other estimates of inequalities. All measures show similar differences in the weekly and cumulative inequalities. There were different patterns seen within each of the regions with higher inequalities seen in the midlands and northern regions, than seen in London and the South. Notably, inequality in cumulative age-standardised mortality rates 28 days after a positive COVID-19 test was very low in the South West and particularly high in the North East and Yorkshire, and the North West and East and West Midlands. Graphs in the supplemental file overlaying the regional data demonstrate particularly well especially low inequality in the South West compared with other regions. However, absolute disparity inequality in weekly age-standardised mortality rates 28 days after a positive COVID-19 test is highest in two large peaks in London, at the start of the pandemic and early in 2021. Inequalities in cumulative rates developed quickly, and while they stabilised and reduced, they did not disappear.

**Figure 2:**
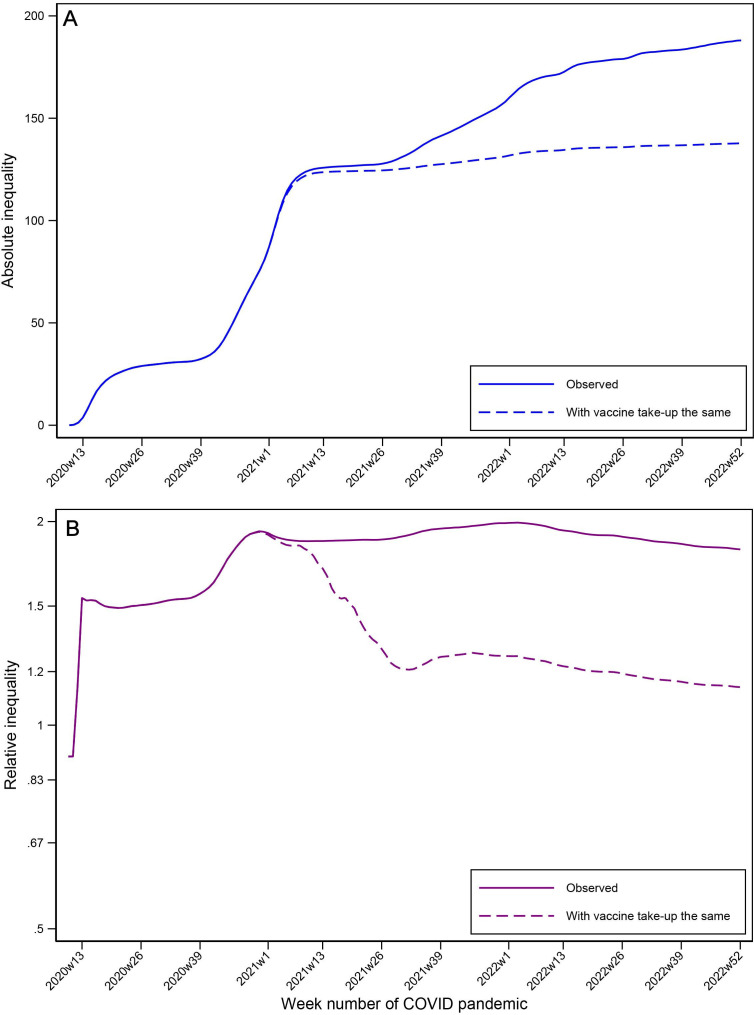
Absolute inequality per 100 000 (top) and relative inequality (bottom) and estimated counterfactual inequalities if vaccination uptake rates in the most deprived areas had been the same as in the least areas in the same region.

The association between the differing uptake of the vaccinations and inequality is shown in figure 2 and table 1. Absolute disparity inequality in age-standardised mortality rates 28 days after a positive COVID-19 test first plateaued and then continued to increase but at a slower rate after the introduction of vaccinations, with relative inequality stabilising. If the vaccination rates in the most deprived areas had been the same as in the least deprived areas, additional absolute disparity inequality (from the start of the vaccination programme) would have been reduced from 118.9 per 100 000 (95% CI 117.0 to 120.7) to 40.2 (95% CI 3.7 to 76.7) and relative inequality (from the start of the vaccination programme) reduced from 1.97 (95% CI 1.92 to 2.02) to 1.30 (95% CI 1.08 to 1.52). The figure shows that even if vaccination rates had been identical, the inequalities that formed quickly at the beginning of the pandemic would not have been completely reversed. During each lockdown, there was a reduction in inequality; though due to the uniformity of the lockdowns on all areas simultaneously, it is not possible to investigate whether the lockdowns directly influenced inequality.

**Table 1:** Log linear model results of absolute inequality (per 100 000) and relative inequality assessing the association of inequality with vaccination and lockdowns (95% CIs)

Time varying covariates	Absolute inequality per 100 000 (ln)		Relative inequality (ln)	
Difference in vaccine factor between least and most deprived	6.15	(1.8–10.5)	2.02	(0.83–3.57)
Fixed covariates				
Lockdown 1	−0.33	(−0.60 to −0.05)	−0.24	(−0.39 to −0.10)
Lockdown 2	−0.87	(−1.30 to −0.44)	−0.34	(−0.57 to −0.12)
Lockdown 3	−0.60	(−0.82 to −0.38)	−0.27	(−0.38 to −0.15)
Adjusting for vaccination				
Lockdown 3	−0.68	(−0.92 to −0.44)	−0.30	(−0.42 to −0.17)

## Discussion

This study provides evidence that the national COVID-19 vaccination strategy in England was associated with a reduction in inequalities in age-standardised mortality rates 28 days after a positive COVID-19 test. However, despite similar uptake at the start of the vaccine rollout, over time, with more doses and as rollout was expanded to more age groups, uptake in the most deprived quintile began to lag behind the least deprived. In this study, we explicitly examined the association between vaccine uptake data from a national COVID-19 vaccination strategy and both *relative and absolute* inequalities in age-standardised mortality rates 28 days after a positive COVID-19 test. We were able to assess population-wide inequalities by using area-level deprivation. An analysis of individual-level vaccination uptake and individual-level COVID-19 mortality, linked with an individual-level socioeconomic status indicator for the entire population, is not possible in England due to data restrictions. Another strength of this research lies in its simplicity. The data used in this study are all publicly available, and our analysis could easily be replicated with the methods used (see online supplemental file 1).

However, several limitations should be considered. First, it is important to note that this is an ecological study. The data reflect the qualities of LTLAs and not the individuals within them. Therefore, our conclusions surrounding vaccination and mortality inequality pertain specifically to population effects rather than an individual effect. Moreover, LTLAs are a relatively large geographic area and population size (most contain 60 000–5 00 000 people), and there may be considerable heterogeneity between neighbourhoods within each LTLA. Relatedly, we had to estimate LTLA age-adjusted deaths from regional age-stratified deaths. Though we perform tests to ensure that these estimates are consistent with the known total number of deaths 28 days after a positive COVID-19 test for each LTLA, it is possible that the age distribution of deaths in each LTLA could differ from what we assume after taking account of the population structure. Additionally, we were unable to disaggregate our analyses by sex due to lack of available data. Furthermore, we do not adjust for differing COVID-19 variants, though some appear to have been less likely to cause severe outcomes. Finally, the three lockdown periods, as the three primary periods (highlighted on figure 1), were applied equally to the whole country, and hence, differences between areas were not possible to disentangle.

Data on age-standardised deaths with COVID-19 on the death certificate and age-stratified figures for LTLAs as the data are no longer released at the LTLA level due to small numbers and associated identification risks. We therefore estimated age-standardised mortality rates 28 days after a positive COVID-19 test at the LTLA level from the published regional level age-stratified mortality figures. Research suggests that the proportion of deaths within 28 days of a positive COVID-19 test which have COVID-19 listed as a factor on the official death certificate declined over as the pandemic progressed into 2022.^27^ However, it remains a good proxy for death from COVID-19 where these data are not available.

Our findings follow indications from the existing literature that overall vaccination uptake is associated with socioeconomic inequalities in COVID-19–related mortality.^16^,^19^ However, in our study, we are additionally able to demonstrate the extent to which inequalities in vaccination uptake may have contributed to inequalities in COVID-19–related deaths. Additionally, we were able to examine a much longer period of vaccination and death data than has previously been studied. This meant that it was possible to observe the role of the vaccine uptake across all three doses and to examine trends beyond the periods of lockdown in 2020 and 2021. One existing study attempted to assess the impact of vaccination on inequalities in mortality in England.^16^ However, this study was only able to assess mortality up until April 2021, likely capturing lockdown effects rather than vaccination.

Our study finds strong evidence that population-wide vaccination for COVID-19 is associated with reductions in mortality inequality. However, it additionally demonstrates that inequalities in both vaccination (especially in the third dose) and mortality remain. Given research on the third dose suggests that it is important for maintaining immunity,^12^ the decline in uptake across the whole population for the third dose and greater inequalities in uptake for this dose is concerning. Future research should examine how to increase uptake in the most deprived communities. These results should be used to inform future vaccine strategies for COVID-19 and future pandemics. Our results indicate a need to emphasise area-level deprivation in future vaccine rollout strategies to reduce inequalities in vaccination uptake and COVID-19 mortality.^28^ This could include tailored strategies in more deprived areas, such as an increased emphasis on community pharmacies.^29^ As those most vulnerable to severe outcomes from COVID-19 and future epidemics are disproportionately located in deprived areas, targeting these areas may also provide the best opportunity to reduce total mortality.^30^

### Conclusions

Our research suggests that population-wide vaccination against COVID-19 is associated with a reduction in inequalities in mortality. However, inequalities formed quickly before vaccination rollout and then persisted. It is therefore necessary to monitor the impact of pandemic responses on inequalities throughout pandemics and to tailor our responses to those who are most vulnerable. Future pandemic responses should be designed with existing inequalities in mind, and vaccination strategies should use a tailored approach to minimise the barriers to uptake for people who are most vulnerable and at risk.

## supplementary material

10.1136/bmjopen-2024-085195 online supplemental file 1

## Data Availability

Data are available in a public, open access repository.
