# Supplementary material for: Ecological study of the association between the English national vaccination programme and area deprivation inequalities in COVID-19 mortality
Source: BMJ Open. 2025 Jan 21;15(1):e085195. doi: 10.1136/bmjopen-2024-085195 (PMC11784416; doi:10.1136/bmjopen-2024-085195)

Supplementary Appendix

Methods

Calculating age-standardised death 28 days after a positive COVID-19 test rate

Age-standardised deaths with COVID-19 on the death certificate and age-stratified figures for LTLAs are no longer released at low area-levels due to the risk of identification.[1,2] The process for calculating age-standardised mortality rates 28 days after a positive COVID-19 test for LTLAs required several steps, as neither age-standardised nor age-separated data are available at the LTLA level. This is problematic as age-specific data are necessary in order to be able to age-standardise the estimates (which is essential in order to compare mortality rates of LTLAs with different age structures). Some limited age standardised COVID-19 mortality data is available online from March 2020 until April 2021. However, this is not sufficient for the purpose of this paper.

To recreate age-standardised mortality rates 28 days after a positive COVID-19 test for the LTLAs, regional age-specific deaths for each date were used. Population data by age for each LTLA was obtained from the Office for National Statistics (ONS)[3] $\mathrm{Pop}_{\mathrm{Age,LTLA}}$. Total daily deaths per LTLA were downloaded for the period March 2020-December 2022 $\mathrm{DailyDeath}_{\mathrm{LTLA}}$. Regional population data were calculated each day by summing the LTLA population by age where there was at least one COVID-19 death $\mathrm{EstDailyPop}_{\mathrm{Age,Reg}}$. Regional daily deaths $\mathrm{DailyDeath}_{\mathrm{Age,Reg}}$ by age were downloaded for the same period and COVID-19 death rates per age were calculated based on regional deaths and summed LTLA populations in areas where a COVID-19 death occurred.

$$\mathrm{RateDeath}_{\mathrm{Age,Reg}} = \frac{\mathrm{DailyDeath}_{\mathrm{Age,Reg}}}{\mathrm{EstDailyPop}_{\mathrm{Age,Reg}}}$$

To calculate the age-specific unknown numbers of deaths 28 days following a positive COVID-19 test an iterative procedure was used that calculated the age-specific values to be consistent with the known daily totals in each LTLA and the known age-specific totals for each region.

The proportion of the daily deaths in a region that were seen in each LTLA (for all ages) were calculated.

$$\mathrm{DailyLTLAtoRegion}_{\mathrm{LTLA}} = \frac{\mathrm{DailyDeath}_{\mathrm{LTLA}}}{\sum_{\mathrm{Age}} \mathrm{DailyDeath}_{\mathrm{Age,Reg}}}$$

STEP 1:

Age-specific deaths were estimated for each LTLA by combining the region age-specific rates with the population of the LTLA (for areas where a death was recorded).

$$\text{Est}_1\text{Death}_{\text{Age,LTLA}} = \text{RateDeath}_{\text{Age,Reg}} \cdot \text{Pop}_{\text{Age,LTLA}}$$

Estimated total daily deaths in an LTLA were then calculated and the proportion of these estimated total deaths to the regional deaths seen estimated adjusted for over / under estimation.

$$\text{EstDailyLTLAtoRegion}_{\text{Reg}} = \frac{\sum_{\text{Age}} \text{Est}_1\text{Death}_{\text{Age,LTLA}}}{\sum_{\text{Age}} \text{DailyDeath}_{\text{Age,Reg}}}$$

These estimates are then used to adjust the daily deaths

$$\text{Est}_2\text{Death}_{\text{Age,LTLA}} = \text{Est}_1\text{Death}_{\text{Age,LTLA}} \cdot \frac{\text{DailyLTLAtoRegion}}{\text{EstDailyLTLAtoRegion}}$$

Additionally, the differences in the regional age specific deaths that are estimated by summing the estimated LTLA deaths versus the observed regional age specific deaths are calculated.

$$\text{EstDeathRate}_{\text{Age,Reg}} = \text{Pop}_{\text{Age,Reg}} \cdot \sum_{\text{LTLA}} \text{Est}_2\text{Death}_{\text{Age,LTLA}}$$

$$\text{EstDailyRateLTLAtoRegion}_{\text{Age,Reg}} = \frac{\text{EstRateDeath}_{\text{Age,Reg}}}{\text{DeathRate}_{\text{Age,Reg}}}$$

This difference is used to adjust the estimated age specific deaths:

$$\text{Est}_3\text{Death}_{\text{Age,LTLA}} = \frac{\text{Est}_2\text{Death}_{\text{Age,LTLA}}}{\text{EstDailyRateLTLAtoRegion}_{\text{Age}}}$$

Then return to step 1 until the estimated deaths by age are consistent with both the daily number of deaths in an LTLA and the regional age specific death rates.

Weekly deaths by age were calculated by summing the daily age specific estimates. Age-standardisation of these estimates was completed by dividing the age group-specific number of deaths for each LTLA by the age-group specific population of each LTLA and then multiplying this by 100,000. This figure was then multiplied by the 2013 European Standard Population and divided by 100,000 to give the standardised rate of death per week for each LTLA.

Zero deaths were assumed in the weeks before the first recorded death in each region.

COVID-19 Testing

The Coronavirus.data.gov website defines a 'positive COVID-19 test' as the following:

"A positive case is identified by a confirmed positive test from a polymerase chain reaction (PCR) test, rapid lateral flow test or loop-mediated isothermal amplification (LAMP) test. Positive rapid lateral

flow test results can be confirmed with PCR tests taken within 72 hours. If this PCR test result is negative, these are removed as cases.” [4]

Lab-based polymerase chain reaction (PCR) COVID-19 testing in England was free and available to the general public via an online form from May 2020[5], until April 1st 2022. After this, COVID-19 PCR tests were mostly limited to clinical settings, testing is therefore unlikely to differ much across deprivation quintiles. Furthermore, it is important to note that the majority of deaths with COVID-19 on the death certificate happened in clinical or care settings and that the change to limiting availability of PCR tests only to clinical settings is unlikely to have dramatically changed our outcome measure or to have led to any differences by geography or socio-economic status.[6]

Index of Multiple Deprivation

The IMD data are provided at the Lower layer Super Output Area (LSOA) level.[7] It was therefore necessary to recalculate the IMD scores to the LTLA level, which is a larger geography. We followed Appendix N of the technical report supplied with the 2019 IMD data[8] which describes the process of aggregating the IMD estimates for larger areas. Based on information available in section 3.8 of the report, we chose to calculate the average of the LSOA IMD ranks within each LTLA. As part of the process for calculating the average rank, population data from mid-2015 estimates[9] (those which were used to produce the 2019 IMD) were used to weight the analysis. From this, a quintile measure was produced, whereby the most deprived LTLAs were assigned one and the least deprived LTLAs were assigned five.

Vaccination rates factor

It is important to undertake an analysis that takes into account that vaccinations were cumulative and that more immunity was incurred (and sustained) with additional vaccinations. Vaccinations were rolled out over a period of time however and as Figure 1 shows, this caused sudden increases in the proportion of the population with one, two or three vaccines. The variable measured as proportion of the population was also then influenced particularly by the third vaccination wave, as the addition of the third vaccine meant that the proportion with just two vaccines then decreased. To analyse each of the doses separately would have caused difficulties, as before the introduction of the third vaccine, two vaccine doses was considered optimal, whereas after the roll out of the third dose, having only two doses was considered suboptimal. To enable the population vaccine level to be appropriately analysed, a factor analysis of the proportion of each population within an LTLA with one vaccination, two vaccinations and three vaccinations was undertaken. The first factor was taken for the analysis. Factor loadings were 0.9391, 0.9762 and 0.7448 with the first factor expressing 100% proportion of the variance.

Inequality estimates

It is important when investigating inequality that more than one measure of inequality is used. In this paper we use three measures that have all be used extensively elsewhere and in papers that investigate COVID-19 inequalities[10-15]. See Table 1 for a description. The diversity index is estimated using age-standardised mortality estimates for the most deprived quintile (quintile 1) divided by the least deprived quintile (quintile 5)[15] with the absolute diversity measured by the difference in the mortality estimates for the most deprived quintile (quintile 1) minus the least deprived quintile (quintile 5). The concentration index and the generalised concentration index are estimated using the rank weighted sum of the age-standardised mortality rates adjusted for region[13,14]Inequality rank is

defined as the quintiles of inequality converted to a 0/1 variable with the quintiles equally distributed. Weekly absolute and relative inequality are estimated using the linear prediction of the relationship between weekly age standardised mortality adjusted for region and the inequality rank[10]. To smooth the relationship and mitigate the zero weeks estimates, we include a rolling three weeks of data. The cumulative absolute and relative inequalities are estimated in the same way.

Table A1

**Observed inequalities**	
Disparity index	A ratio variable calculated by dividing the mortality rate estimates for the most deprived quintile by the least deprived quintile.
Absolute disparity inequality	The difference between mortality rate estimates of the most deprived quintile and least deprived quintile.
Concentration index	The concentration index captures the extent to which mortality differs across LTLAs ranked by deprivation quintile. The concentration index is twice the area between the concentration curve and the 45° line indicating no relationship between deprivation and mortality. It is a value which increases with greater concentration of mortality among the more deprived quintiles.
Generalised concentration index	An absolute inequality measure from the concentration index. Calculated by multiplying the concentration index by the mean mortality rate.
**Modelled inequalities**	
Absolute inequality	The estimated linear effect of deprivation on mortality rate across all five quintiles.
Relative inequality	The relative risk of the deprivation in the least to the most deprived estimated from the linear effect of deprivation as above.

The code used for the analyses in this paper is available from the authors upon request.

Supplementary References

1. Office for National Statistics. Deaths due to COVID-19 by local area and deprivation. 2021. https://www.ons.gov.uk/peoplepopulationandcommunity/birthsdeathsandmarriages/deaths/datasets/deathsduetocovid19bylocalareaanddeprivation (accessed 05/10/2022.

2. Office for National Statistics. Area type definitions Census 2021. 2023. https://www.ons.gov.uk/census/census2021dictionary/areatypedefinitions#:~:text=tier%20local%20authorities-,Lower%20tier%20local%20authorities%20provide%20a%20range%20of%20local%20services,(including%20City%20of%20London) (accessed 20/09/2023.

[dataset] 3. Office for National Statistics. Estimates of the population for the UK, England, Wales, Scotland and Northern Ireland. 2022. https://www.ons.gov.uk/peoplepopulationandcommunity/populationandmigration/populationestimates/datasets/populationestimatesforukenglandandwalesscotlandandnorthernireland14/02/2023).
4. UK Health Security Agency. Metrics documentation: Cumulative cases by publish date. 2023. https://coronavirus.data.gov.uk/metrics/doc/cumCasesByPublishDate#england (accessed 16/02/2023.
5. Department of Health and Social Care. Everyone in the United Kingdom with symptoms now eligible for coronavirus tests. 2020.
6. Office for National Statistics. Deaths due to COVID-19, registered in England and Wales. 2021. https://www.ons.gov.uk/peoplepopulationandcommunity/birthsdeathsandmarriages/deaths/datasets/deathsduetocovid19registeredinenglandandwales2020 (accessed 29/07/2024.
[dataset] 7. Department for Communities and Local Government [DCLG]. The English Indices of Deprivation. 2019. https://www.gov.uk/government/statistics/english-indices-of-deprivation-2019 (accessed 07-09-2022.
8. McLennan D, Noble S, Noble M, Plunkett E, Wright G, Gutacker N. The English Indices of Deprivation 2019: Technical report, 2019.
[dataset] 9. Office for National Statistics. Lower layer Super Output Area population estimates (supporting information). 2021. https://www.ons.gov.uk/peoplepopulationandcommunity/populationandmigration/populationestimates/datasets/lowersuperoutputareamidyearpopulationestimates (accessed 07/12/2022.
10. Mackenbach JP, Kunst AE. Measuring the magnitude of socio-economic inequalities in health: an overview of available measures illustrated with two examples from Europe. *Social science & medicine* 1997; **44**(6): 757-71.
11. Mackenbach JP, Stirbu I, Roskam A-JR, Schaap MM, Menvielle G, Leinsalu M, Kunst AE. Socioeconomic inequalities in health in 22 European countries. *New England journal of medicine* 2008; **358**(23): 2468-81.
12. Blakely T, Disney G, Atkinson J, Teng A, Mackenbach JP. A Typology for Charting Socioeconomic Mortality Gradients: “Go Southwest”. *Epidemiology* 2017; **28**(4): 594-603.
13. O'Donnell O, O'Neill S, Van Ourti T, Walsh B. Conindex: estimation of concentration indices. *The Stata Journal* 2016; **16**(1): 112-38.
14. Asada Y, Grignon M, Hurley J, et al. Trajectories of the socioeconomic gradient of mental health: Results from the CLSA COVID-19 Questionnaire Study. *Health Policy* 2023; **131**: 104758.
15. Lawton R, Zheng K, Zheng D, Huang E. A longitudinal study of convergence between Black and White COVID-19 mortality: A county fixed effects approach. *The Lancet Regional Health–Americas* 2021; **1**.

Figure 1a: Line graph showing weekly relative disparity index on the left hand y axis, the average proportion of the population of the LTLAs within the quintiles vaccinated with one, two and three doses on the right hand y axis, and weekly average proportion of the whole population of each LTLA in quintiles one (most) and five (least) vaccinated by dose.

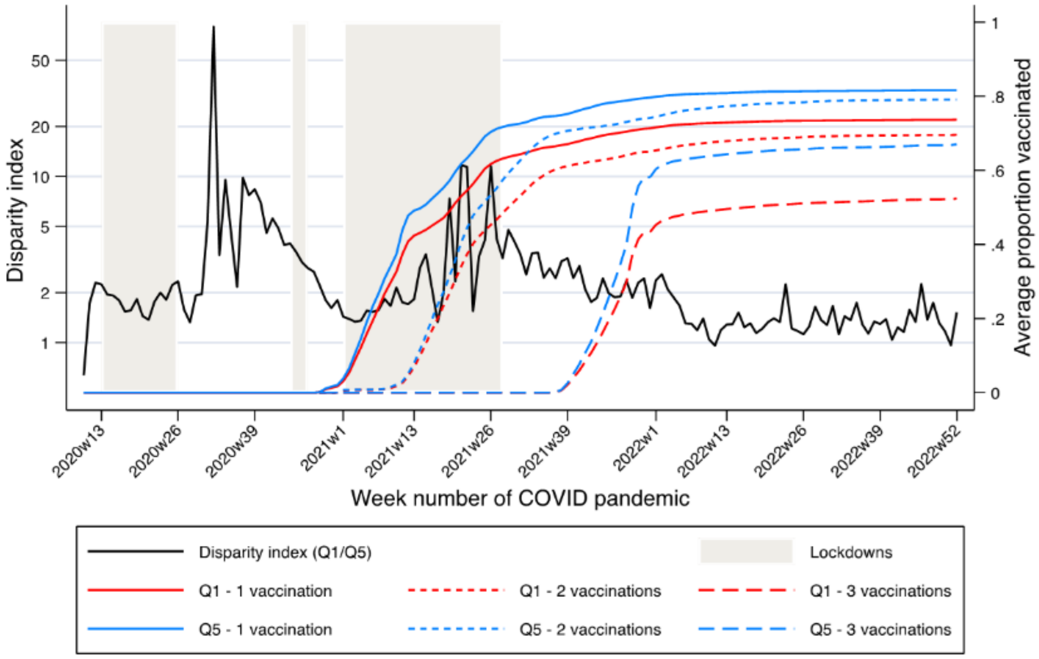

Figure 1b: Relative difference (disparity index) between the most and least deprived areas in cumulative age standardised mortality 28-days after a positive COVID-19 test rates and average proportion vaccinated over time

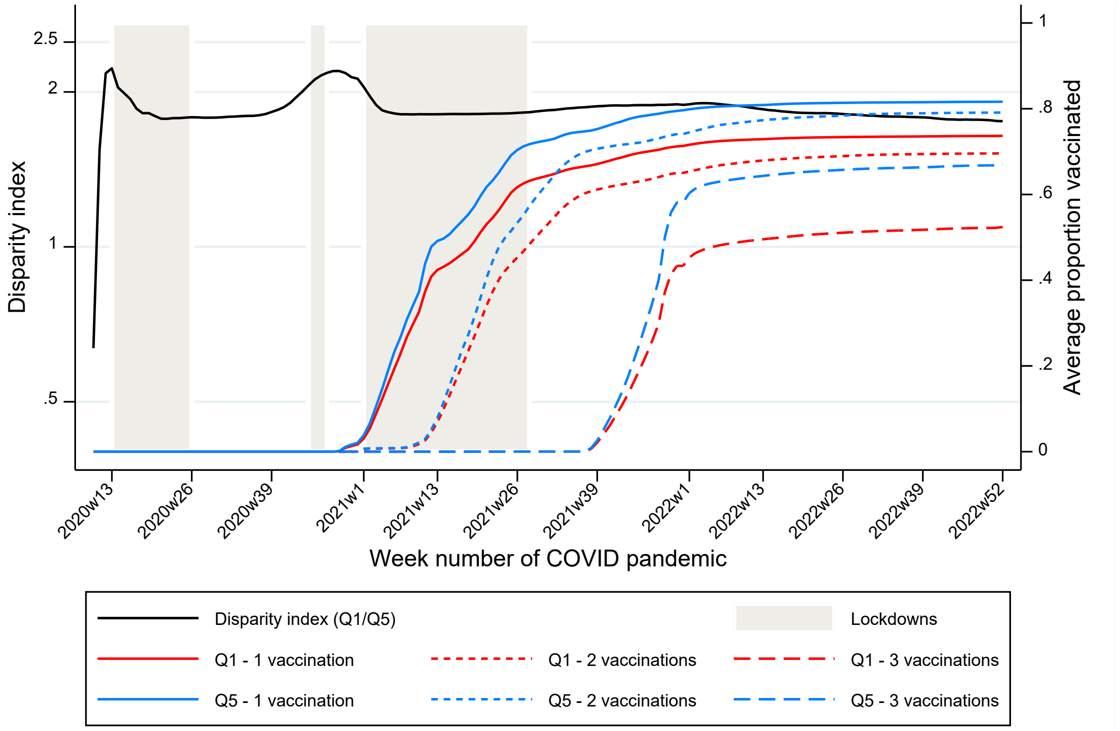

Figure 1c: Weekly absolute disparity inequality between the most and least deprived areas in age standardised mortality 28-days after a positive COVID-19 test rates and average proportion vaccinated over time

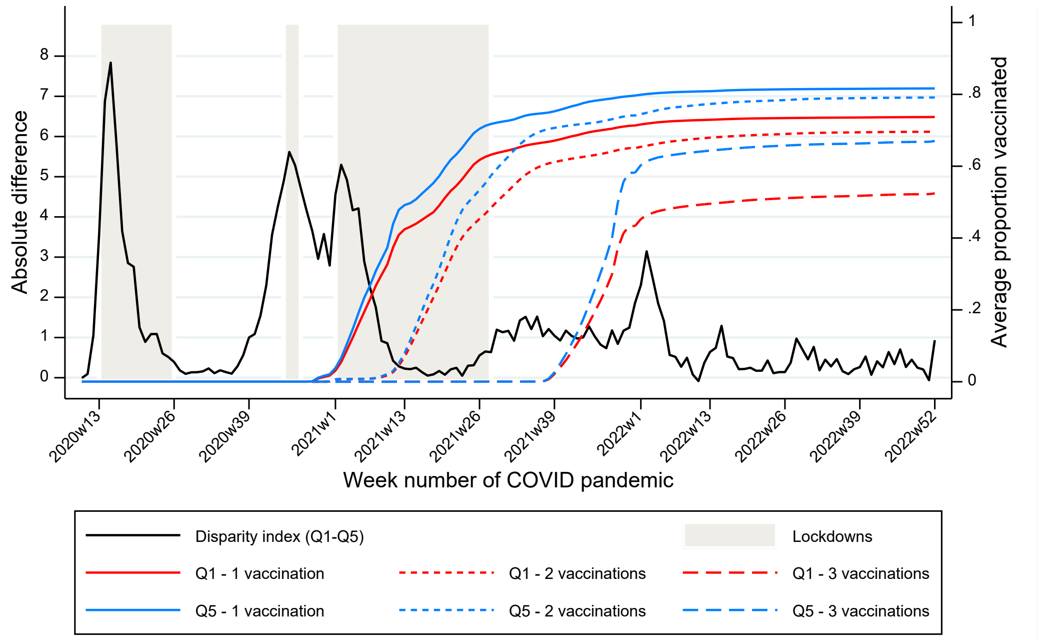

Figure 2a: Disparity index between most and least deprived areas in weekly age standardised mortality 28 days after a positive COVID-19 test rates, overall and for each region

Weekly disparity between the most deprived and least deprived are highly variable. When the least deprived has zero deaths and the most deprived see one or more, the rates are indicated with a purple dropline above the line of equality. A similar dropline is plotted when the least deprived see one or more deaths but the most deprived see none.

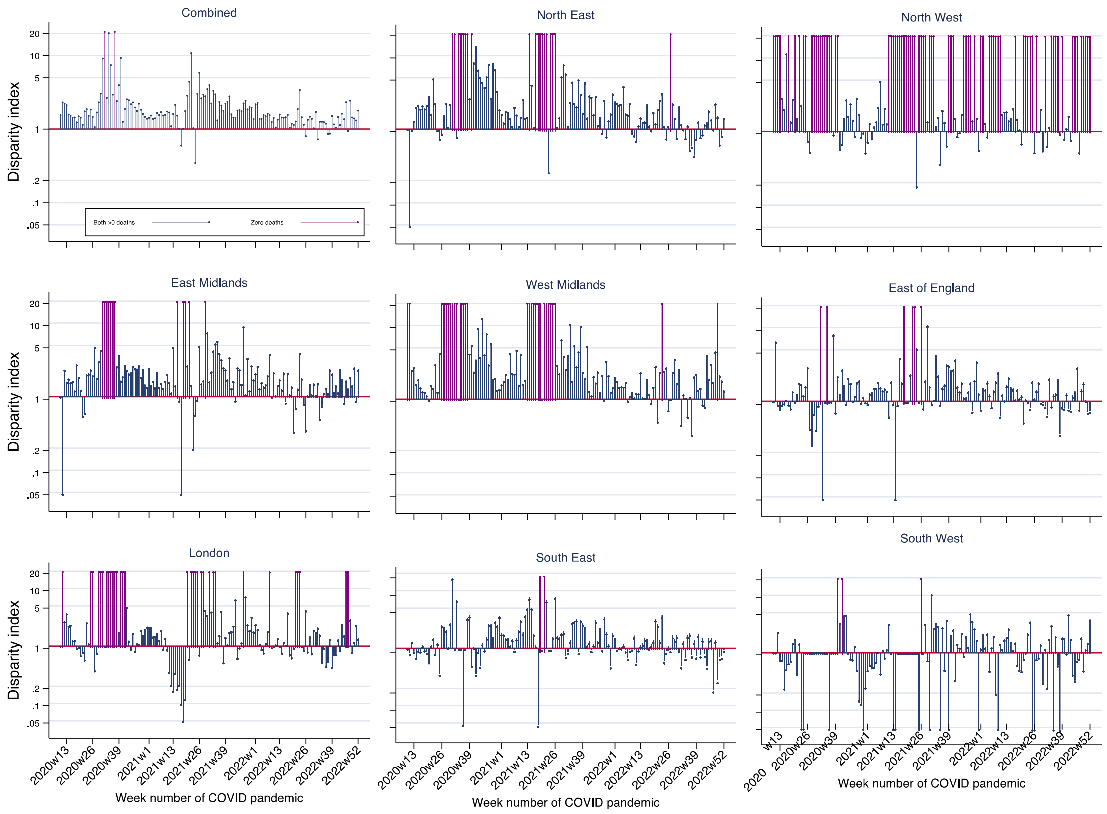

Figure 2b: Disparity index between most and least deprived areas in cumulative age standardised mortality 28 days after a positive COVID-19 test, overall and for each region

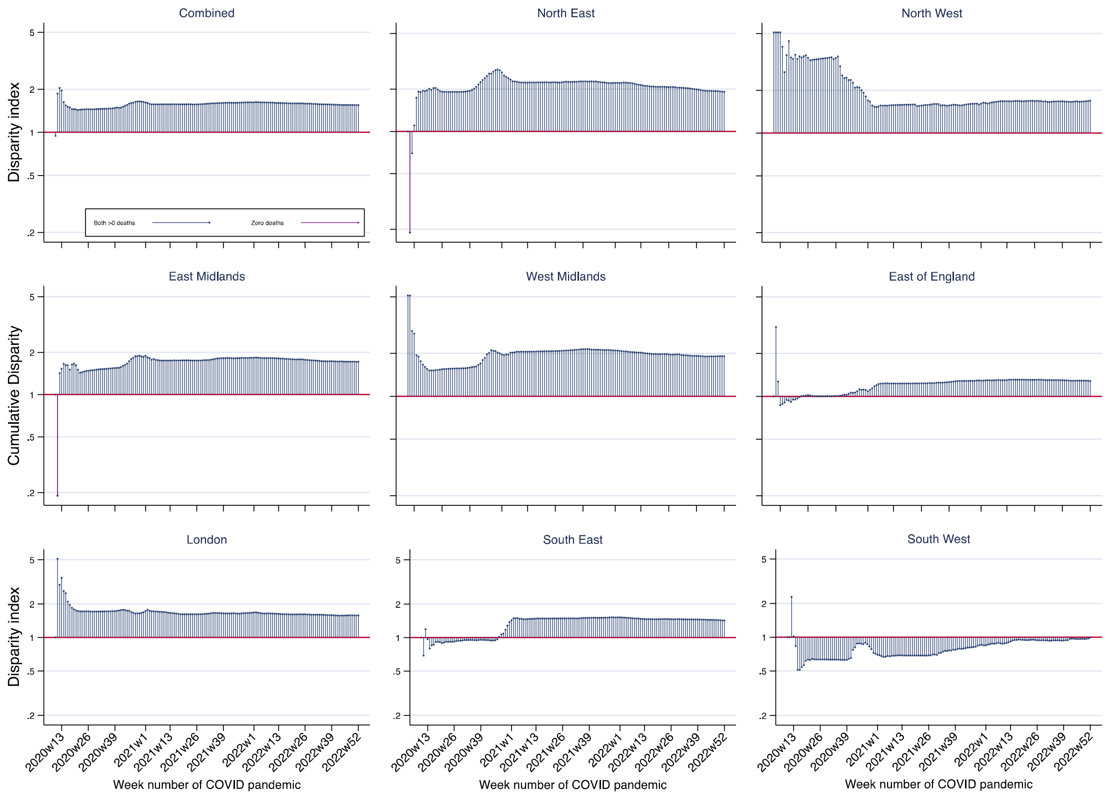

Figure 3a: Concentration index across deprivation in weekly age standardised mortality 28 days after a positive COVID-19 test rates, overall and for each region

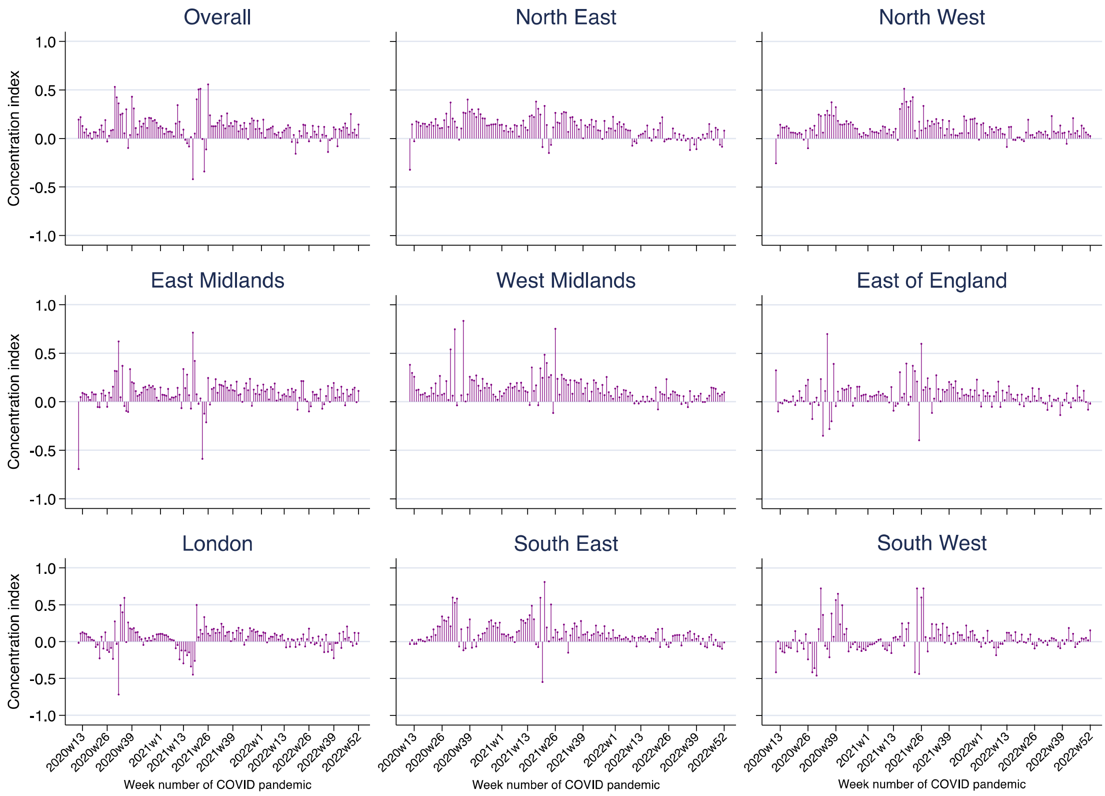

Figure 3b: Generalised concentration index across deprivation in weekly age standardised mortality 28 days after a positive COVID-19 test rates, overall and for each region

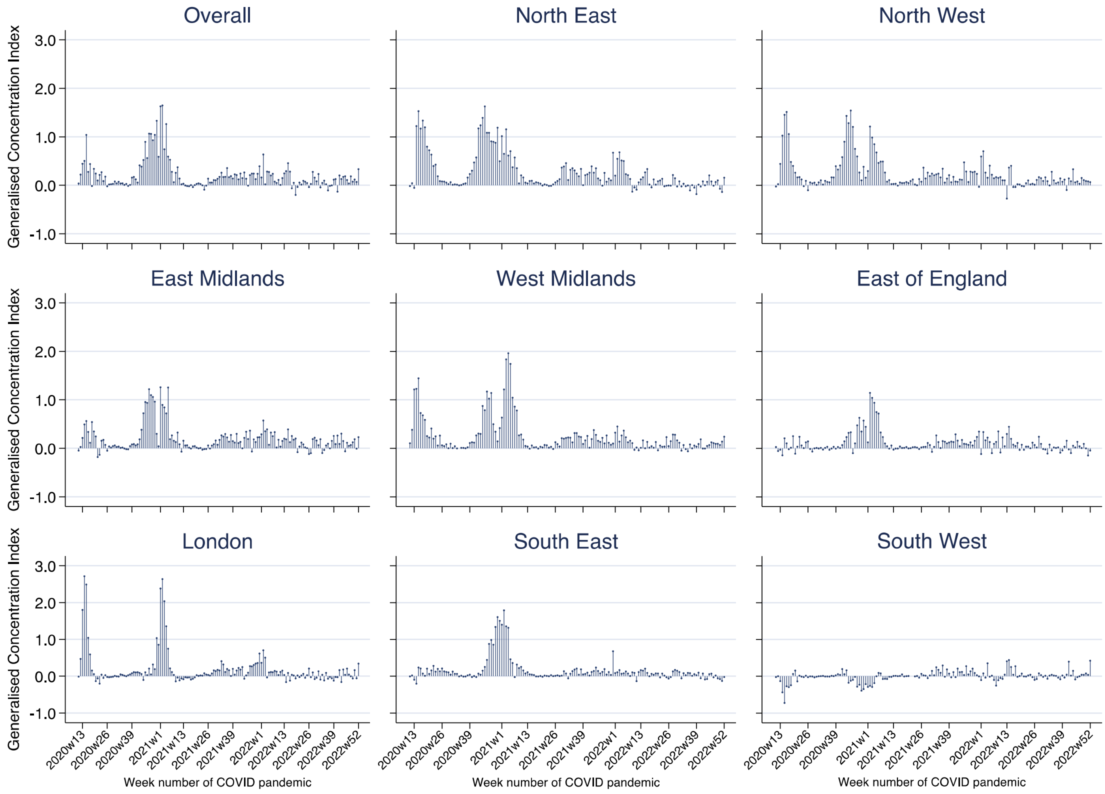

Figure 3c: Concentration index across deprivation in cumulative age standardised mortality 28 days after a positive COVID-19 test rates

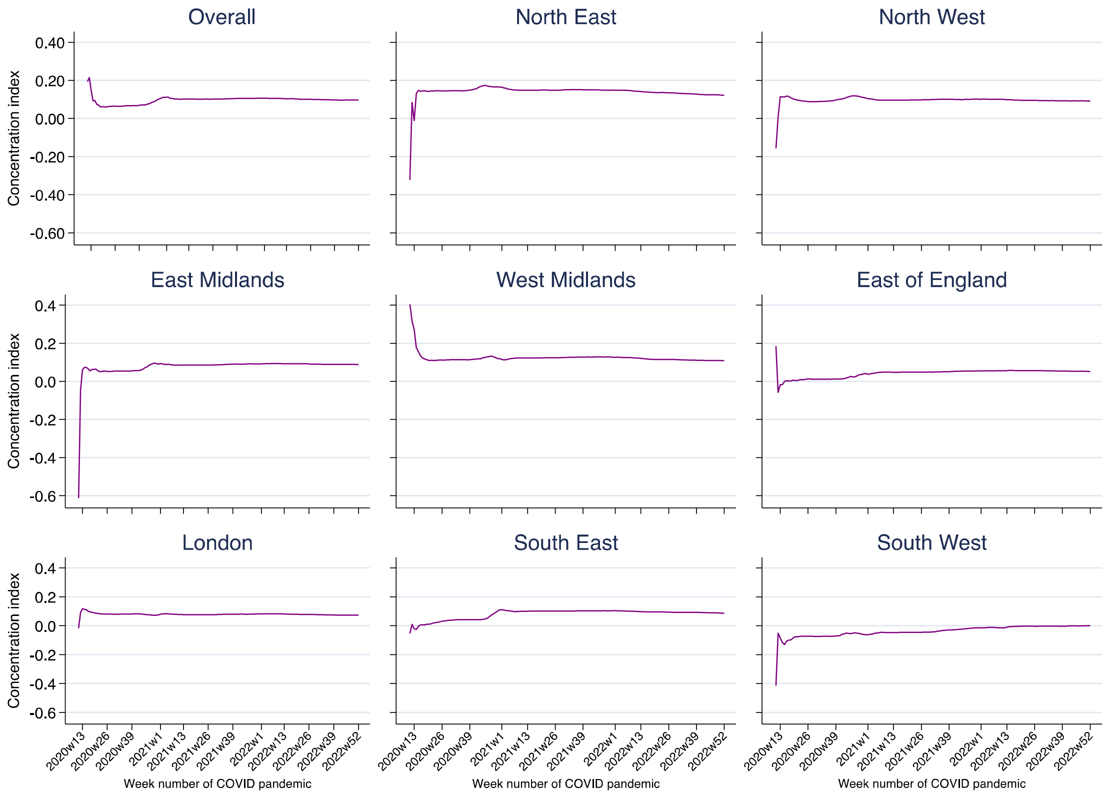

Figure 3d: Generalised concentration index across deprivation in cumulative age standardised mortality 28 days after a positive COVID-19 test rates

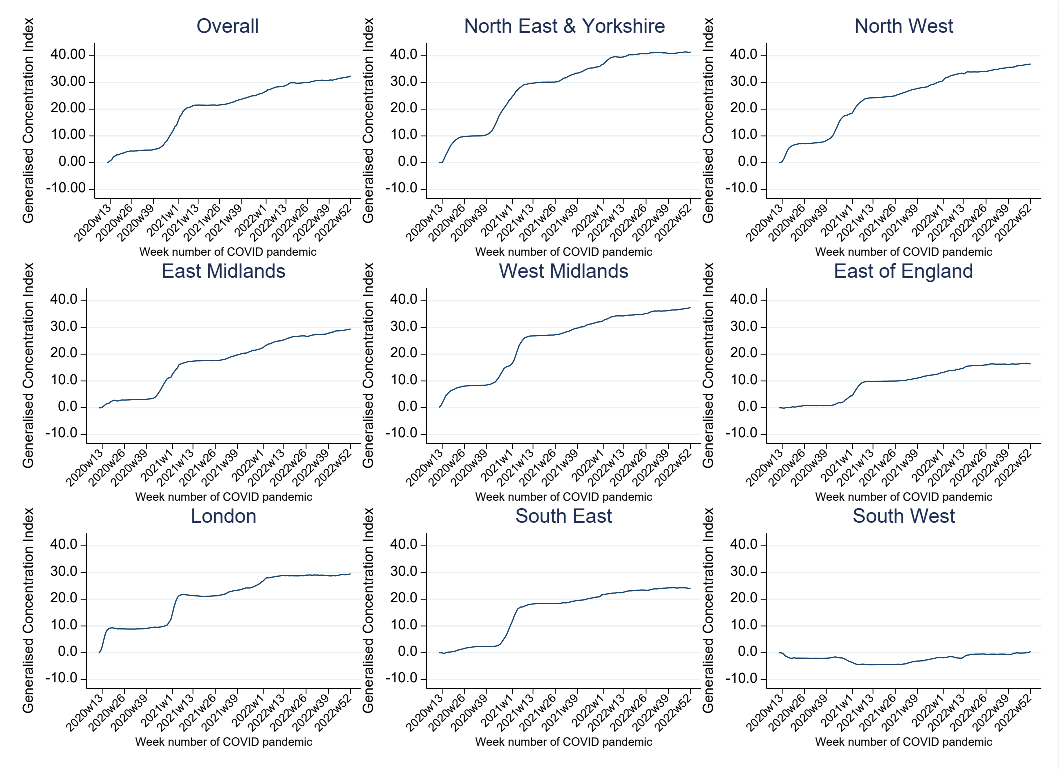

Figure 4a: Dropline of absolute disparity inequality in weekly age standardised mortality 28 days after a positive COVID-19 test rates, overall and by region

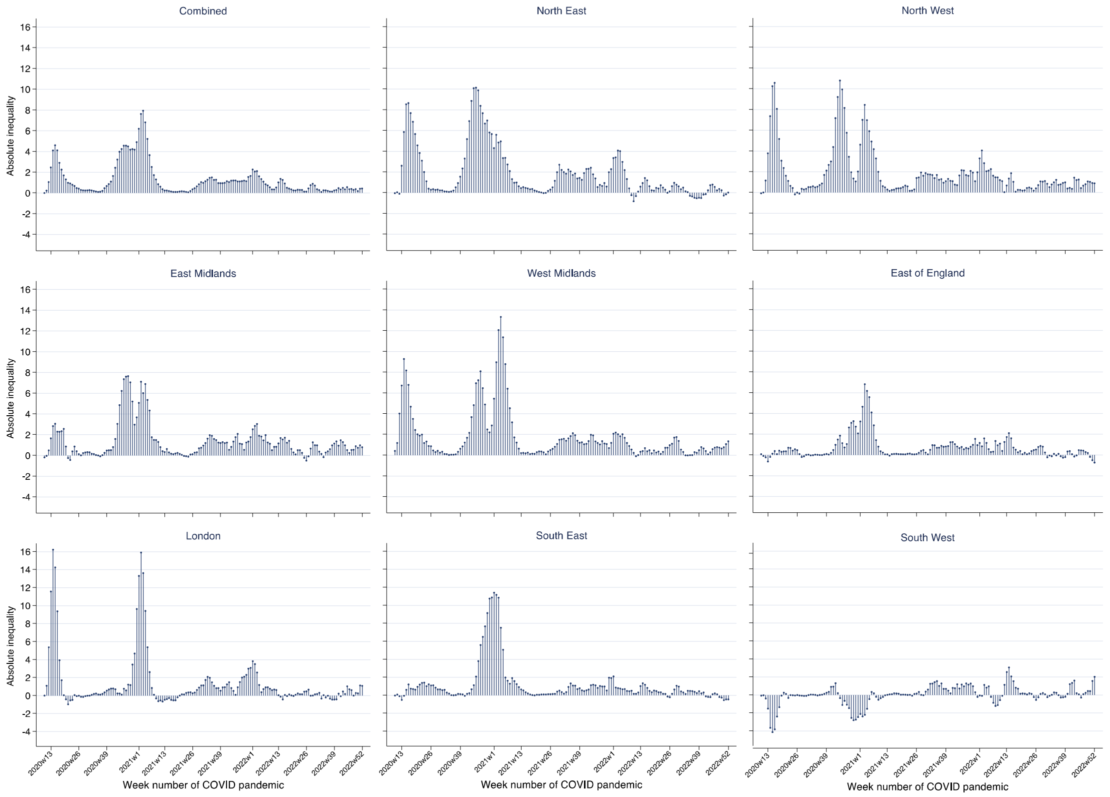

Figure 4b: Dropline of relative inequality in weekly age standardised mortality 28 days after a positive COVID-19 test rates, overall and by region

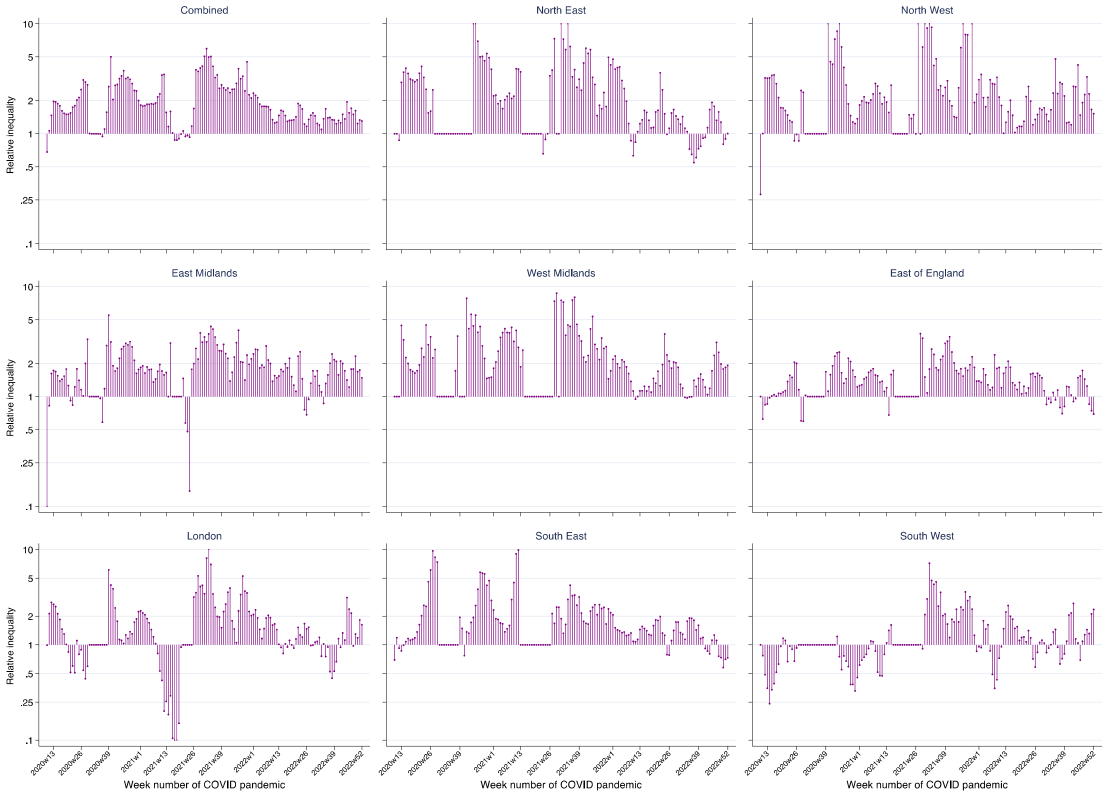

Figure 6a: Absolute disparity inequality in cumulative age standardised mortality 28 days after a positive COVID-19 test rates, overall and by region

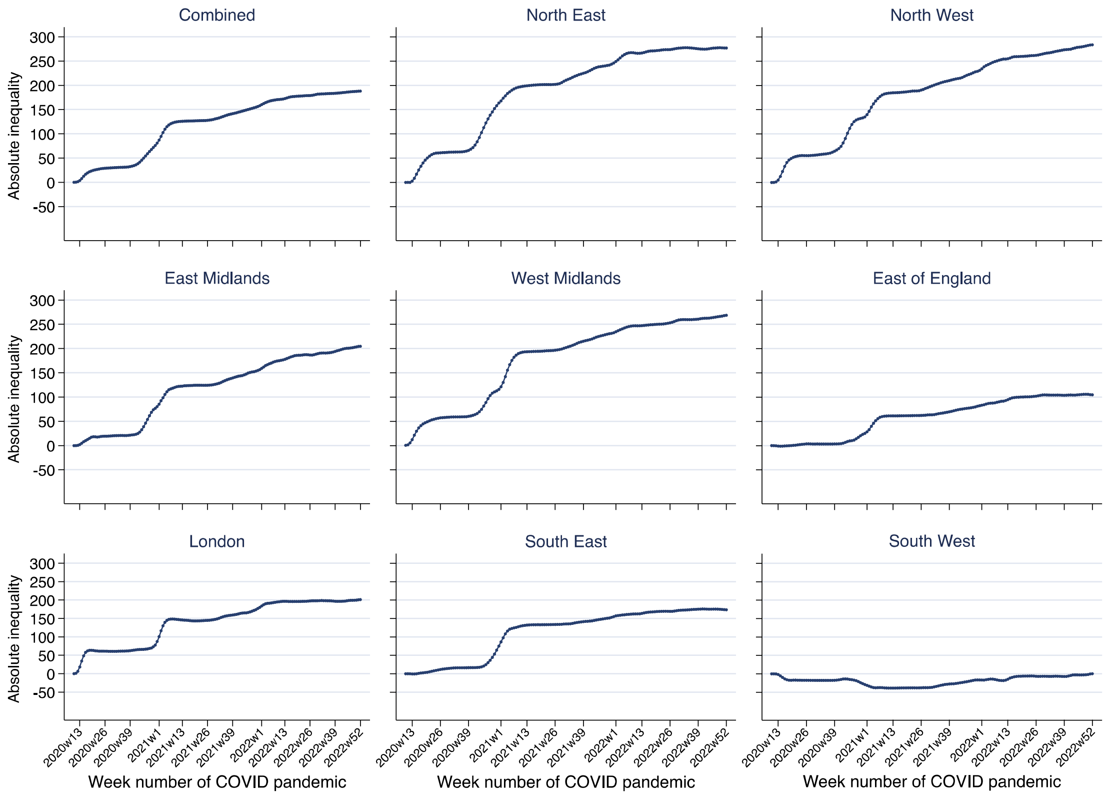

Figure 6b: Relative inequality in cumulative age standardised mortality 28 days after a positive COVID-19 test rates, overall and by region

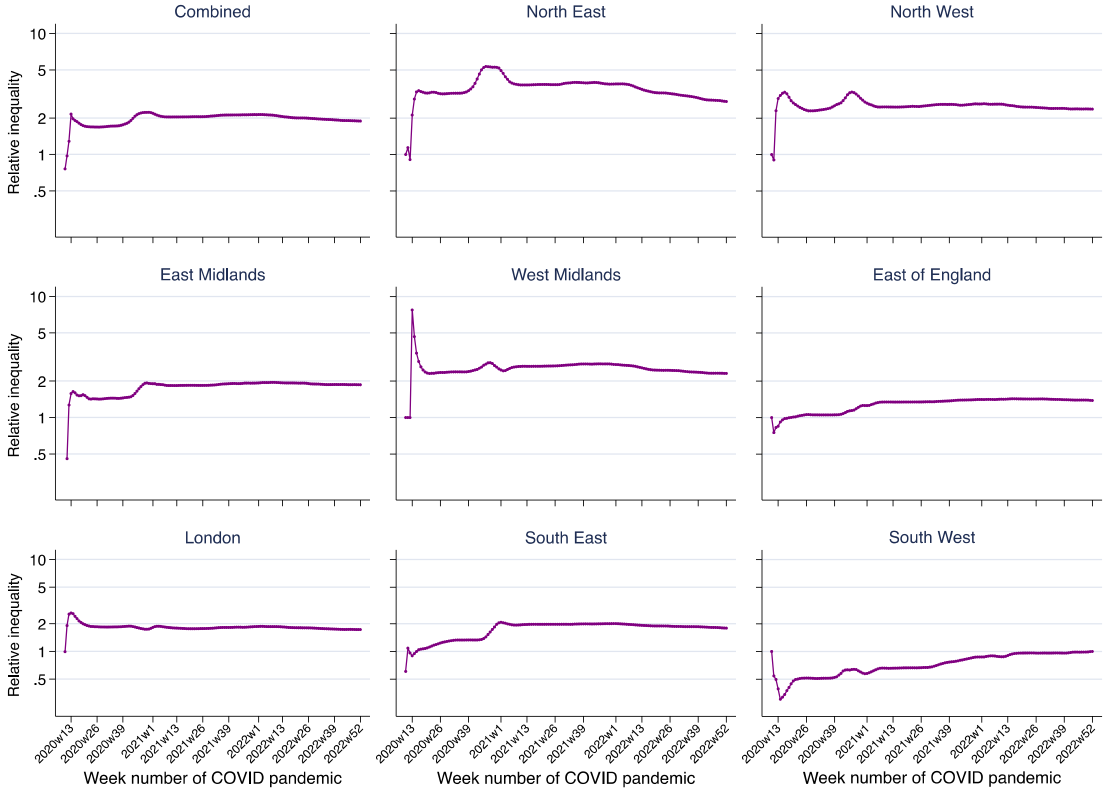

Graphs of regional differences

Figure 7a: Generalised concentration index in weekly age standardised mortality 28 days after a positive COVID-19 test rates, region comparison

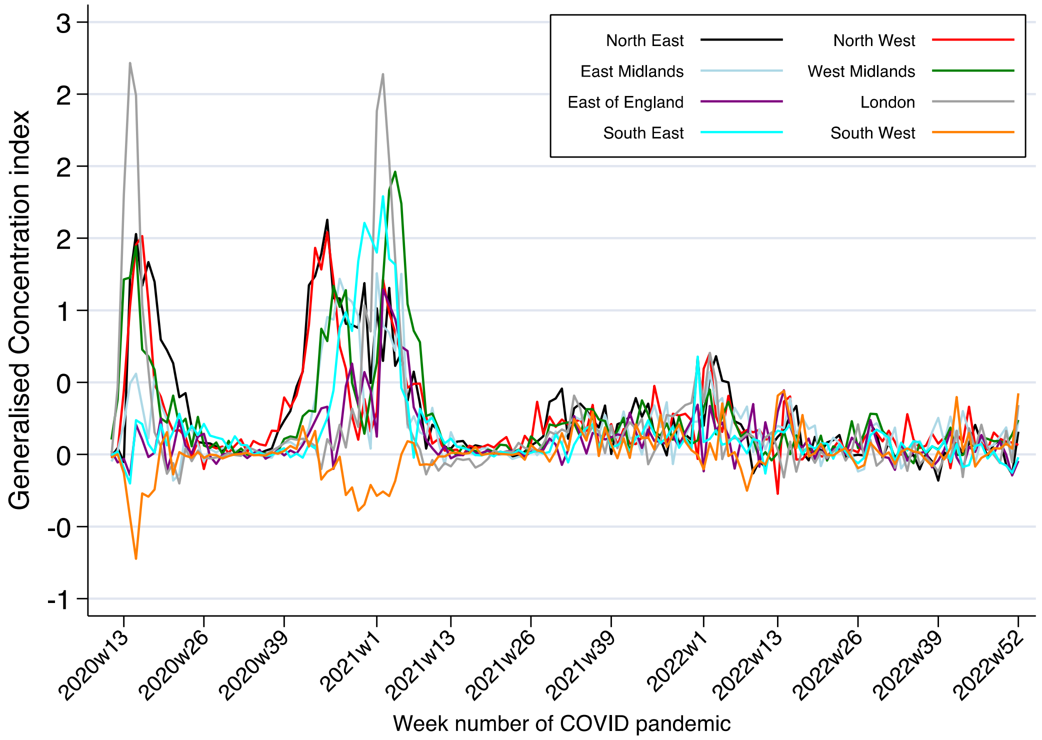

Figure 7b: Concentration index in weekly age standardised mortality 28 days after a positive COVID-19 test rates, region comparison

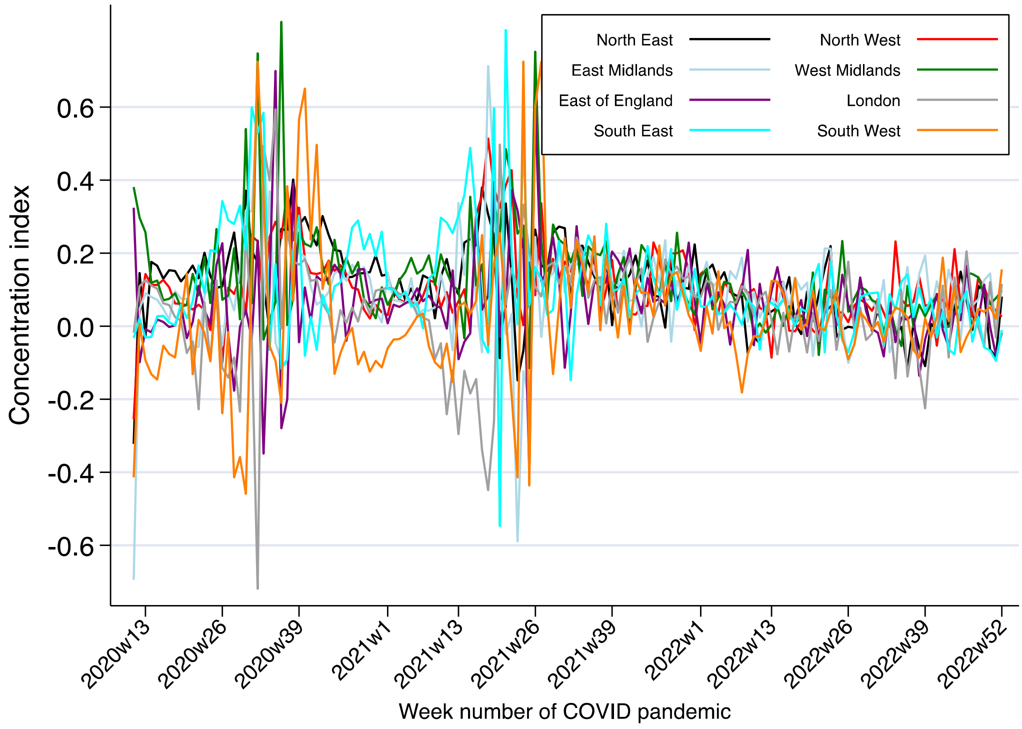

Figure 8a: Generalised concentration index in cumulative age standardised mortality 28 days after a positive COVID-19 test rates, region comparison

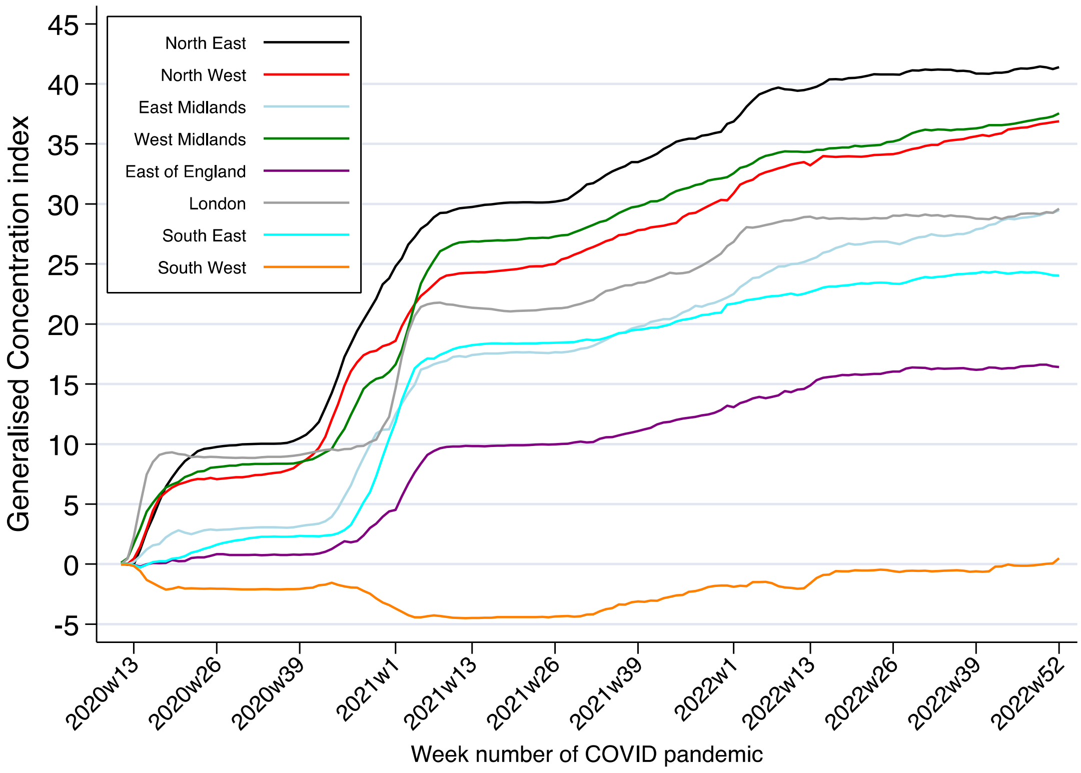

Figure 8b: Concentration index in cumulative age standardised mortality 28 days after a positive COVID-19 test rates, region comparison

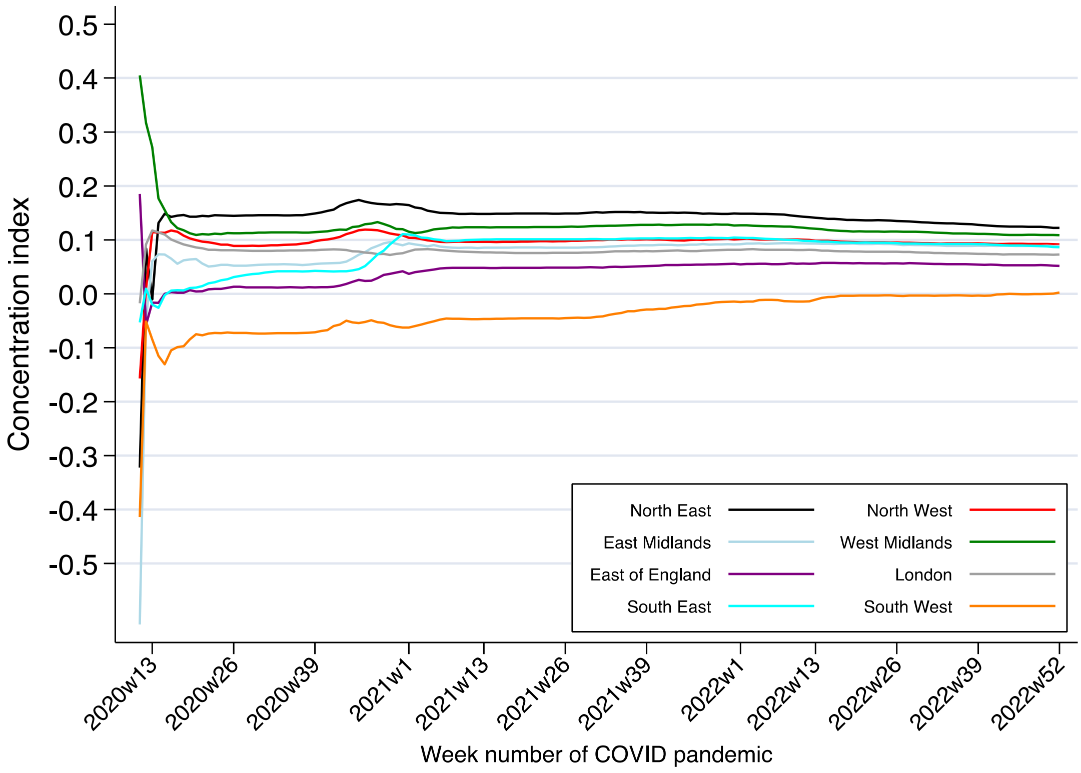

Figure 9a: Absolute disparity inequality in weekly age standardised mortality 28 days after a positive COVID-19 test rates, region comparison

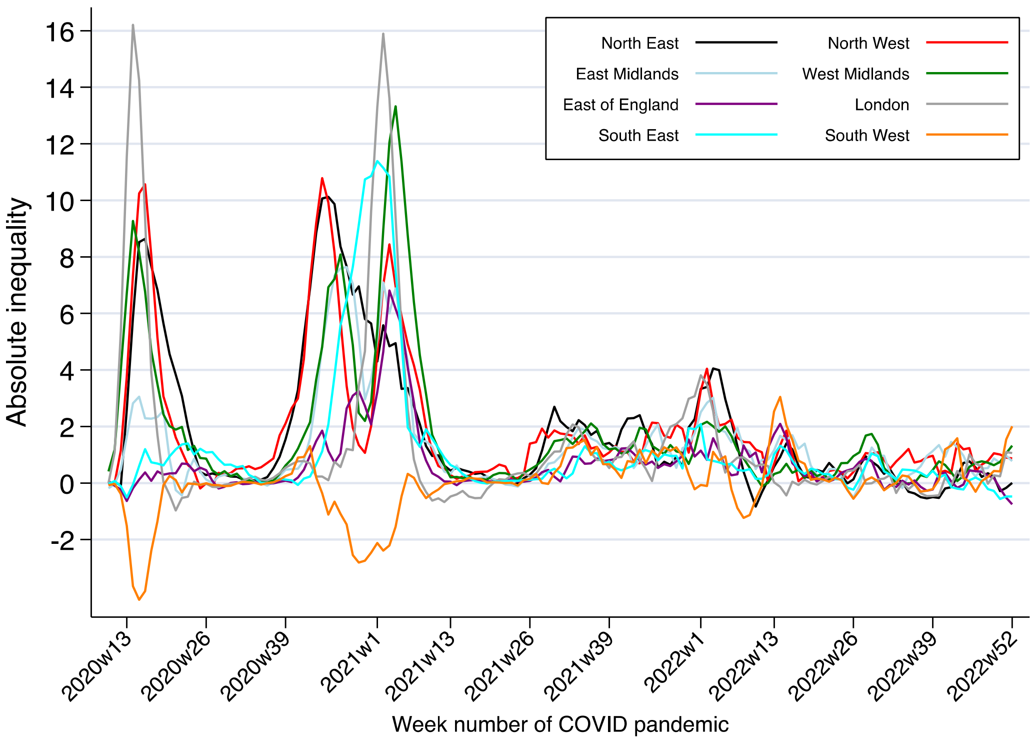

Figure 9b: Relative inequality in weekly age standardised mortality 28 days after a positive COVID-19 test rates, region comparison

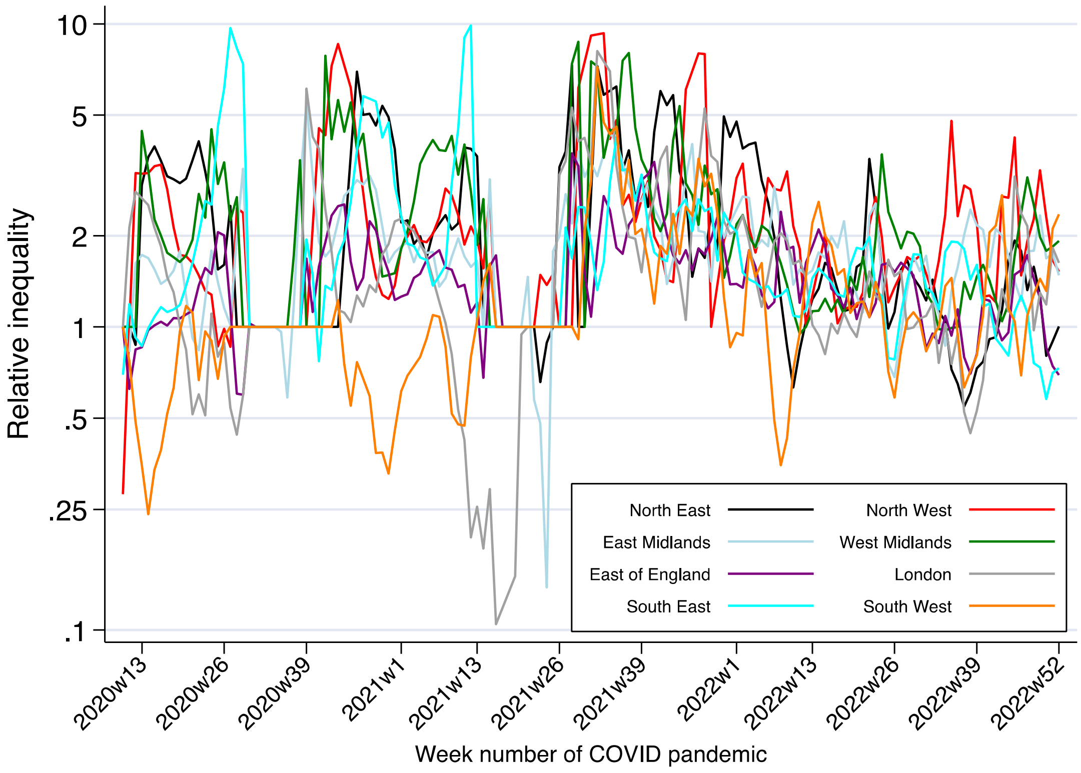

Figure 10a: Absolute disparity inequality in cumulative age standardised mortality 28 days after a positive COVID-19 test rates, region comparison

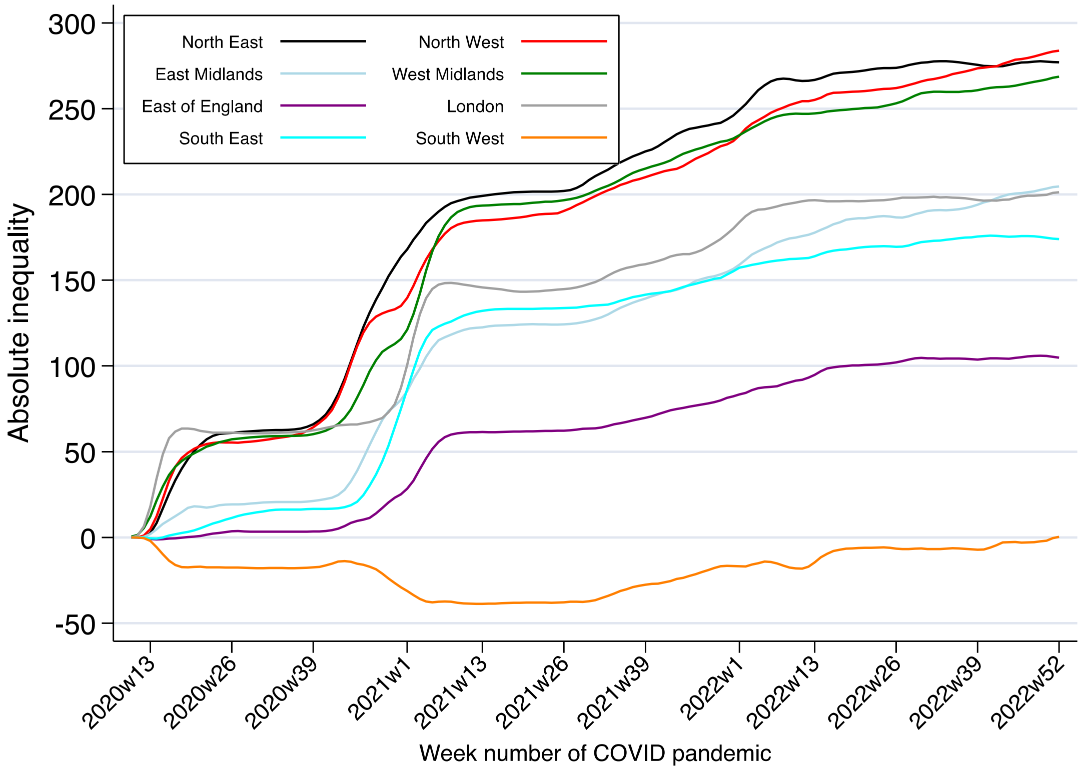

Figure 10b: Relative inequality in cumulative age standardised mortality 28 days after a positive COVID-19 test rates, region comparison

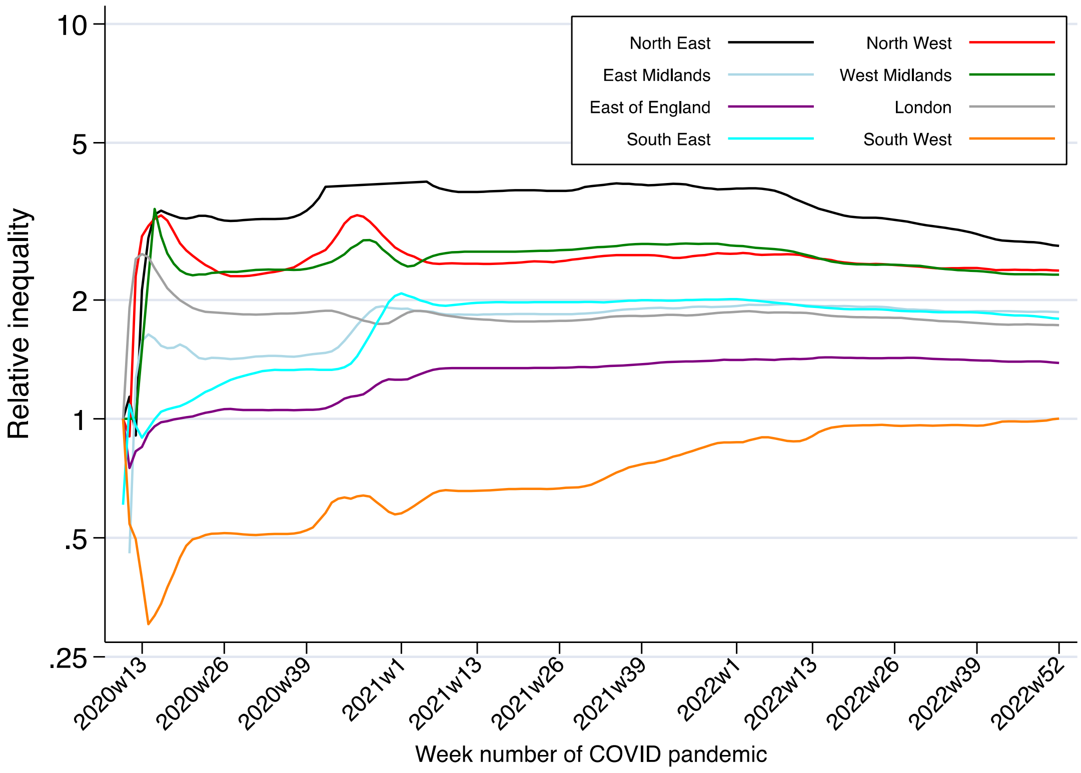

Supplement: online supplemental file 1 [file bmjopen-15-1-s001.pdf]
