# Supplementary material for: Ecological study of the association between the English national vaccination programme and area deprivation inequalities in COVID-19 mortality
Source: BMJ Open. 2025 Jan 21;15(1):e085195. doi: 10.1136/bmjopen-2024-085195 (PMC11784416; doi:10.1136/bmjopen-2024-085195)
Supplement: Supplementary file 2 [file bmjopen-2024-085195.reviewer_comments.pdf]

PEER REVIEW HISTORY

BMJ Open publishes all reviews undertaken for accepted manuscripts. Reviewers are asked to complete a checklist review form and are provided with free text boxes to elaborate on their assessment. These free text comments are reproduced below.

ARTICLE DETAILS

Title (Provisional)

An ecological study of the association between the English national vaccination programme and area deprivation inequalities in COVID-19 mortality

Authors

Bennett, Natalie; Bambra, Clare; Sinclair , David; Todd, Adam; Matthews, Fiona

---

VERSION 1 - REVIEW

**Reviewer**	**1**
**Name**	**Bandyopadhyay, Siddhartha**
**Affiliation**	**University of Birmingham, The Department of Economics**
**Date**	**19-Mar-2024**
**COI**	**None**

This is a really interesting question and one that appears to have been understudied. It is on the whole clear and reads well. Some clarification on a few issues would help enrich the analysis further.

While individual level data is not available, it's not clear from the main body why a lower level of aggregation was not possible i.e. LSOA level data could not be used. It would be useful to mention what variables were not made available at lower levels of aggregation which would leave the authors with no other choice but I could not see it being explicitly mentioned. Some of the calculations in the Appendix make this clearer but a mention in the main text pointing out that data availability (at least using publicly available sources) makes analysis below this level infeasible.

p.8 I am glad that the endogeneity problem is mentioned but the possible source for endogeneity is not really clearly explained. Is the potential source that vaccination rates and death rates both vary by time which leads to a spurious correlation between them? I think a more detailed explanation as well as how is the remedy suggested reducing endogeneity?

**Reviewer**	**2**

Name **Oberndorfer, Moritz**

Affiliation **University of Helsinki**

Date **08-Apr-2024**

COI **None**

This is a valuable study showing that vaccination against COVID-19 reduced inequalities in COVID-19 related mortality in the UK. The introduction and discussion sections were concise and balanced. Below, the authors will find some suggestions to improve the clarity of their analysis and presentation of their results. I believe it is highly useful to estimate how mortality and inequalities in mortality may have been different had the most deprived areas higher vaccination up-take (e.g., vaccination up-take of the least deprived areas, 10% higher up-take, or other useful and realistic scenarios). This part of the study may deserve a bit more space in the main text (or appendix if space is lacking).

This study will be even more useful for the research community trying to estimate how hypothetical measures on the vaccination up-take can reduce inequalities in the mortality burden of COVID-19 and future pandemics if the estimands of interest and the according methods are clearly described in the manuscript. I hope my comments can support the authors in improving their highly relevant manuscript.

Strengths and limitations:

Comment 2:
"We assessed population-wide inequalities by using area-level deprivation as a measure of socioeconomic status"

I am not sure if the authors are doing themselves a conceptual favour if they use area-level deprivation as a measure of (individual-level?) socioeconomic status. To me, it would be fair to present these as inequalities along area-level (multiple) deprivation (of LTLA). In other words, the conceptual leap from area-level multiple deprivation to socioeconomic status is not necessary. Area-level inequalities are valuable in their own right. Please consider this suggestion.

Comment 3:

"This enabled us to model the difference between actual inequalities vaccination rates and hypothetical inequalities if uptake in the most deprived areas had been more similar to the least deprived"

You might have forgotten an "in" here. (actual inequalities *in* vaccination rates). If by "actual" you mean observed, you could consider using "observed inequalities" for clarity.

Comment 4:

"We use publicly available data to conduct the analysis and also make our code available, supporting reproducibility efforts"

Unfortunately, I did not find a link to the code. This would have helped me to provide a more informed review of the analysis. If I have missed a link in the manuscript, kindly refer me to it.

Introduction:

It's a short and informative introduction. I have no suggestions to improve it.

Methods

Study design and data sources

Comment 5:

Please consider describing the unit of analysis (LTLAs) in more detail regarding average (SE) /median/ range of population size already in the method section.

Comment 6:

"An overall estimate for vaccination was created using factor analysis of the proportion who have one, two or three vaccines[22]. Factor loadings are shown in the Supplementary Appendix."

It is not obvious to me why the authors use factor analysis here. There is also no explanation for this approach in the given reference 22. Please consider providing a rationale for this factor analysis to the reader for easier interpretation of the measure. I am also not sure what exactly is meant by "overall estimate of vaccination". Why do the authors choose to estimate a factor underlying the proportions of LTLA population over simply using the readily interpretable observed proportion of the LTLA population that was vaccinated? Please also explain how this variable (whichever you decide to go for) is used in your analysis.

Statistical analysis

Comment 7:

"The impact of lockdowns and differences between vaccine take-up on absolute and relative inequalities of COVID-19 mortality were investigated using generalised log-linear models. Vaccination impact was estimated using factor loadings for the proportion who have one, two or three vaccines over time[22]. Generalised log-linear models were used to investigate absolute and relative inequality and the relationship with vaccine take-up and lockdowns. To reduce issues of endogeneity (where the mortality rate and therefore the amount of inequality detectable, and vaccination rates all vary over time) the mean age-standardised absolute and relative inequalities (logged) in each region were regressed on mean vaccine update[26]."

I appreciate that the authors have limited space to describe their methods. However, I would suggest being more precise here and formulate a specific estimand (or multiple) of interest instead of referring to "the impact of…" or "relationship with …". That would help the reader to understand what exactly the authors seek to estimate right away. Further, please provide a reason for taking the log of your outcomes or any other variables. I have tried to find an explanation in the reference 26, but I could only find this part in the method section of reference 26:

"When assessing factors that were time-varying, such as the policy and behavioural responses, the dependent variable was the mean of the log-transformed daily rates, a specification that can be shown to mitigate bias that occurs when using cumulative measures, despite the fact that the

hypothesised mechanism connecting the dependent and independent variables is immediate." (Bollyky et al. 2023)

This is not followed up by any further explanation or references. Apologies, but it is not obvious to me what is gained by transforming these variables. These analytical decisions would be easier to understand if the authors specified targeted estimates as mentioned above. Currently, it is difficult appreciate your analytical approach if the estimands of your study are not sufficiently described.

Comment 8:

"As lockdowns were undertaken uniformly across all areas, a generalised mixed log-linear model with random effects for region on the mean age-standardised weekly log absolute and relative inequalities over time was used to investigate the impact of lockdowns."

This sounds interesting, but I am not sure what exactly the goal of this analysis was. Please consider providing more detail here.

The Appendix contains further information about the statistical analysis. I appreciate that a more detailed description of the analysis was probably moved to the appendix to stay within the word count limit. This is a very valid solution, but I suggest making sure that, where relevant, the authors refer to the details provided in the appendix. Currently, the information provided under "Statistical analysis" in the Supplementary Appendix is not linked with the main text through explicit references (eg., "see appendix page X for a more detailed explanation").

Results

Comment 9:

"Figure 1 – Line graph showing weekly relative inequality on the left hand y axis, and weekly average proportion of the whole population of each LTLA in quintiles one (most) and five (least) vaccinated by dose."

Please consider also explaining the right-hand vertical axis in the figure caption of Figure 1.

Comment 10:

"There were different patterns seen within each of the regions with higher inequalities seen in the midlands and northern regions, than seen in London and the South. "

Please consider describing these patterns in more detail with reference to the respective figures.

Comment 11:

"The impact of the different uptake of the vaccinations is shown in Figure 2 and Table 1. Absolute inequality first plateaued then continued to increase but at a slower rate after the introduction of vaccinations, with relative inequality stabilising."

For clarity, I suggest describing the results with "inequality in mortality 28 after a positive COVID-19 test" or "COVID-19 related mortality" instead of simply referring to inequality. Since inequalities in

vaccination up-take is also of interest in this study, such clarity might avoid confusing the two inequalities.

Comment 12:

Lastly, there are many figures in the supplementary appendix which are not mentioned in the main text. Even if the authors lack the space to describe all their results, it may be worth linking these additional figures to the main text and justify their presentation.

Discussion

The discussion is concise and balanced. I do not have any comments or suggestions.

Reviewer 3

Name Welsh, Jennifer

Affiliation Australian National University , National Centre for Epidemiology and Population Health, Research School of Population Health

Date 10-Apr-2024

COI None.

This manuscript has potential to contribute to be a useful paper that contributes important new knowledge. However, there needs to be more clarify in the methods and results before it is suitable for publication.

Major points in the methods:

• The first paragraph of the methods belongs in the introduction.

• The abstract says that the study is of adults but no information on this is included in the methods.

• Given that this is an ecological study, the two primary measures need to be described as population measures, e.g. rather than deaths within 28 days of a positive test, it needs to be "age-standardised 28-day COVID-related mortality rates". The other outcome, "vaccination doses" isn't clear at all. Proportion of the population who received 1, 2 and 3 doses?

• Their measure of vaccination uptake is not clear at all. The statement on page 7 line 57 "produced a cumulative proportion vaccinated measure" seems to suggest that the main measure is the proportion having received 1, 2, and 3 doses at 26 December 2022? And how this relates to LTLAs I'm not sure? Other places refer to average proportion in the quintile?

• It's unclear to me what went into the factor analysis – or why this was done – and what this measured was used for. Could the authors please clarify?

• The deprivation index includes a measure of health. What implications does this have for the findings? This needs to be incorporated into the discussion in some capacity because by

my relatively quick thinking, this would underestimate inequalities in vaccine uptake between deprivation areas (i.e. more deprived areas have greater proportion of people in poor health, who had higher uptake of vaccines) but it might overestimate inequalities in mortality (more deprived areas have greater proportion of sicker people, who are at greater risk of mortality once infected).

• I couldn't follow the methods used to estimate age-standardised mortality rates in the supplementary material (more comments below). Additionally, given that this is the primary outcome, at least some of the detail on how this was estimated needs to go into the main text.

• The authors need to add their methods for estimating the counterfactual scenario of what inequalities in mortality rates would have been had vaccination uptake been the same across areas. How do they define "the same". In the supplementary file, it says if most deprived quintile had uptake the same as in least deprived quintile, but this needs to be in the main text.

• Given that this is aggregated, whole-of-population data, how do the authors interpret the confidence intervals?

Supplementary file:

• How is a region different to a LTLA? As someone who isn't familiar with the English data, I was confused by this.

• How was age operationalised for these analyses? Single year of age or 10-year age groups? Or something else?

• Throughout the text, can the authors clarify whether they are estimating death rates or death counts?

• Can the authors clarify throughout the text if they are referring to all-cause or COVID-related deaths?

• Page 19 line 24, is "data" a typo and should it say "date"? I struggled with that first sentence.

• Page 19 line 36, what does the "(A) refer to?

• Page 19, line 41, "to calculate the age-specific unknown numbers…" of what? Number of COVID-19 related deaths?

• The equations are not intuitive and seem to use "age" a bit inconsistently. Furthermore, unless I really misunderstood, the use of subscripts for LTLA and region also used quite inconsistently.

• There is only one heading "Step 1", which implies other steps, but no other headings for different steps?

• I'm not sure that I follow, but if I understood correctly, the method used to get age-specific death rates for each LTLA imply that each LTLA has the same mortality as the "region" – is this interpretation correct? What evidence is there that this assumption is true? Or will it all average out in the end given that LTLAs make up the region?

• The authors talk about the estimates being adjusted for "over/ under estimation" (page 20, line 5), what does this mean? How was this done?

• It isn't clear to me why the factor analysis was done or what went into the analysis? What does "100% proportion" refer to?

• Page 21, line 34 "inequality rate is defined as the quintiles of inequality converted to a 0/1 variable with the quintiles equally distributed". I'm not sure what this sentence means – could the author clarify? A dummy variable for each quintile? Equally distributed based on what, population within each LTLA area or equal number of LTLAs within each quintile?

• Why adjust for region? How do you interpret the results when adjusted for region?

• I'm confused as to what their measures of inequality were. They seem to imply a difference between absolute and relative measures and the diversity index and the concentration index, but the diversity index looks like a relative measure to me (it looks like relative rate). I'm not sure what they modelled when they talk about relative and absolute measures?

• On a related note, where they refer to absolute measures, the authors need to add the measure and the scale. What is the measure of the absolute inequality in Figure 2? Rate difference per 100,000?

• The statistical analysis section of the supplementary file needs to go into the main text, and needs to be edited for clarity. What was the outcome in these models and what where the exposures in the model? Why adjust for region? These models seem to be modelling the association between 28-day COVID mortality rates and lockdowns, which where not part of the original aims? Also, if lockdown applied universally (i.e. no variation in the measure between areas) what is this model achieving?

More minor points in the methods:

• Can the authors add more detail on the LTLAs in England? Are they defined by geographical boundaries (i.e. England broken in 309 areas of roughly the same size) or population size? How many people on average live in these areas? This was in supplementary material but needs to go into the methods.

Major points in the results:

• The figures seem to have headings embedded in the images and in text on the page. Suggest just one.

• In Figure 1, I would suggest reporting the absolute inequalities. Absolute differences are where inequalities play out in the real world. Plus, there are (what I assume to be) relative

rates of ~50 in that figure. A RR of 50 is truly enormous and suggests a lot of uncertainty in the estimate. The underlying data are needed to interpret what's going on behind those big estimates.

• The headings in Figure 2 are very non-descript and need to be updated. What are their measures of absolute and relative inequality?

• Table 1 is also quite unclear, including the heading. Absolute inequality measured in what scale? The CIs seem quite wide given the nature of the data, are they correct?

• Page 9, line 37-38, the authors refer to "new mortality" which isn't a great way to describe what they are looking at (all deaths are new deaths). Can the authors revise?

• Can the authors say what they found rather than describing what figures exist in the supplementary file?

• The region-stratified analyses show quite a lot of variation in findings. E.g. the south west seems to buck the trend and have least disadvantaged areas having higher mortality rates relative to the more disadvantaged areas – can the authors comment on that? Is there anything to learn from different approaches taken by the different regions?

• The purple lines in figure 2a exaggerate the points where the data are sparse. It might be better to either not present this figure, or to leave the purple areas completely blank and say that the RRs can't be estimated. And again, there are some really enormous RRs on that figure, which makes me think that the underlying data are quite uncertain.

Limitations:

• What happened to COVID-19 testing over the time period? Are there estimates of what proportion of cases tests were capturing over time? And importantly, did this differ by socioeconomic position? What are the implications for bias of missing positive cases, particularly if ascertainment differs by socioeconomic position?

• The authors note that they have conducted an ecological study but don't really address what specific limitations this type of study design has? I'm concerned about this, particularly given that health is included in the definition of deprivation.

• The authors have had to estimate age-standardised mortality at the LTLA level, which is the primary outcome, but don't discuss what assumptions they've made in doing so, or the implications for bias this creates.

On language:

• We can't determine "the effect" of something on something else with an ecological study. The authors need to revise the manuscript, including the title, to remove causal language.

• The future directions and conclusions are not particularly related to the findings of the study, could they be revised.

• There are a few typos and points where important commas are missing. Could the authors revise for clarity.

VERSION 1 - AUTHOR RESPONSE

Reviewer 1

1. While individual level data is not available, it's not clear from the main body why a lower level of aggregation was not possible i.e. LSOA level data could not be used. It would be useful to mention what variables were not made available at lower levels of aggregation which would leave the authors with no other choice but I could not see it being explicitly mentioned. Some of the calculations in the Appendix make this clearer but a mention in the main text pointing out that data availability (at least using publicly available sources) makes analysis below this level infeasible.

We thank Reviewer 1 for highlighting this lack of clarity. Our choice of LTLA level was in part driven by the lack of outcome data at small-area scales, as well as the LTLA scale being meaningful for local public health decision making. Age-separated death data had to be estimated from a regional level (as only total mortality was provided at the LTLA level due to identification risks). We therefore felt LTLA was the lowest geographical scale we could disaggregate the data to with some confidence. In the text, we provide further detail:

We employ the LTLA scale in order that meaningful geographic inequalities could be estimated, whilst balancing data availability and sufficient mortality data. Further information on data availability and scale constraints is provided in the appendix.

2. p.8 I am glad that the endogeneity problem is mentioned but the possible source for endogeneity is not really clearly explained. Is the potential source that vaccination rates and death rates both vary by time which leads to a spurious correlation between them? I think a more detailed explanation as well as how is the remedy suggested reducing endogeneity?

Yes you are correct, the vaccination rates and the mortality rates will be correlated with time and therefore time invariant analyses were used. If we were to model the deaths on the weekly vaccination proportions that would result in biased estimates of the association. The method used to deal with this is the one explained in detail in the Supplement of the paper referenced (Bollyky TJ, Castro E, Aravkin AY, et al. Assessing COVID-19 pandemic policies and behaviours and their economic and educational trade-offs across US states from Jan 1, 2020, to July 31, 2022: an observational analysis. The Lancet 2023; 401(10385): 1341-60.)

Reviewer 2

1. I am not sure if the authors are doing themselves a conceptual favour if they use area-level deprivation as a measure of (individual-level?) socioeconomic status. To me, it would be fair to present these as inequalities along area-level (multiple) deprivation (of LTLA). In other words, the conceptual leap from area-level multiple deprivation to socioeconomic status is not necessary. Area level inequalities are valuable in their own right. Please consider this suggestion.

   We appreciate this issue being brought to our attention and remove any reference to using deprivation as a proxy for SES in the text.

2. You might have forgotten an "in" here. (actual inequalities in vaccination rates). If by "actual" you mean observed, you could consider using "observed inequalities" for clarity.

   We thank reviewer two for highlighting this and edit the sentence to be the following:

   This enabled us to model the difference between observed inequalities in vaccination rates and hypothetical inequalities if uptake in the most deprived areas had been more similar to the least deprived.

3. Unfortunately, I did not find a link to the code. This would have helped me to provide a more informed review of the analysis. If I have missed a link in the manuscript, kindly refer me to it.

   The code link was commented on in error. The analysis was undertaken within Stata and therefore not readily useable to all individuals. We have therefore added a comment to the code being available on request.

4. Please consider describing the unit of analysis (LTLAs) in more detail regarding average (SE) /median/range of population size already in the method section.

   To provide further detail, we include the following:

   The size of LTLA populations ranges from 41,000 (Rutland) to 1,158,000 (Birmingham) with a mean of 194,000 persons (SD 130,000, SE 7,600) and a median of 149,000 persons.

5. It is not obvious to me why the authors use factor analysis here. There is also no explanation for this approach in the given reference 22. Please consider providing a rationale for this factor analysis to the reader for easier interpretation of the measure. I am also not sure what exactly is meant by "overall estimate of vaccination". Why do the authors choose to estimate a factor underlying the proportions of LTLA population over simply using the readily interpretable observed proportion of the LTLA population that was vaccinated? Please also explain how this variable (whichever you decide to go for) is used in your analysis.

   Determining the efficacy of vaccination in a given area is complicated by the three-dose regime recommended for most people in the UK. Individuals cannot get two doses unless

they have one, nor can they get three before they have two. Undertaking analyses for each number of doses received would cause reverse relationships each time a new dose wave was undertaken as two doses, whilst beneficial initially, subsequently became less beneficial than three once the third dose became available. Creating one variable that indicates the vaccine load of the population enables appropriate analysis of these three variables (% with one, two or three doses). In order to improve clarity, we have changed 'overall estimate of vaccination' to 'population vaccination load'. In addition, we add further justification for the choice of this method in the main text:

We do this so that the complex process of vaccine role out is combined so that a measure of the total population vaccinated at each time point is included in the model whilst balancing model parsimony. We refer to this measure as the population vaccination load.

6. I appreciate that the authors have limited space to describe their methods. However, I would suggest being more precise here and formulate a specific estimand (or multiple) of interest instead of referring to "the impact of…" or "relationship with …". That would help the reader to understand what exactly the authors seek to estimate right away. Further, please provide a reason for taking the log of your outcomes or any other variables. I have tried to find an explanation in the reference 26, but I could only find this part in the method section of reference 26: "When assessing factors that were time-varying, such as the policy and behavioural responses, the dependent variable was the mean of the log-transformed daily rates, a specification that can be shown to mitigate bias that occurs when using cumulative measures, despite the fact that the hypothesised mechanism connecting the dependent and independent variables is immediate." This is not followed up by any further explanation or references. Apologies, but it is not obvious to me what is gained by transforming these variables. These analytical decisions would be easier to understand if the authors specified targeted estimates as mentioned above. Currently, it is difficult appreciate your analytical approach if the estimands of your study are not sufficiently described.

   We agree that this is currently unclear and have now clarified that the aim of the study is to investigate whether relative and absolute inequality in COVID-19 mortality increased or decreased in association with the England COVID-19 vaccination program. Modelling in log is standard for rates, the analysis here looks at the relationship of vaccine usage in mortality. The relative inequality is just a covariate within a standard log linear model. We add:

   This study therefore aims to determine whether relative and absolute inequality in COVID-19 mortality decreased associated with the COVID-19 vaccination program in England.

7. "As lockdowns were undertaken uniformly across all areas, a generalised mixed log-linear model with random effects for region on the mean age-standardised weekly log absolute and relative inequalities over time was used to investigate the impact of lockdowns." This sounds interesting, but I am not sure what exactly the goal of this analysis was. Please consider providing more detail here.

   We appreciate this lack of clarity being brought to our attention. The two main population changes influencing COVID mortality were the vaccination programme and the national lockdowns. Here we were investigating the lockdowns as a comparison to the vaccine effect.

Undertaking the random effects adjusts for the heterogeneity of each region. We have added the following:

Random effects for regions allowed the model to account for variation in our outcomes at the regional level, for example due to differences in population clustering, movement patterns and adoption of social distancing behaviours.

8. The Appendix contains further information about the statistical analysis. I appreciate that a more detailed description of the analysis was probably moved to the appendix to stay within the word count limit. This is a very valid solution, but I suggest making sure that, where relevant, the authors refer to the details provided in the appendix. Currently, the information provided under "Statistical analysis" in the Supplementary Appendix is not linked with the main text through explicit references

   We thank Reviewer 2 for inviting us to make clearer links between our main text and appendix materials. Though we do flag the presence of the further detail we provide in the appendix in the main text ("*A comprehensive definition of the outcome measure and process for calculating age-standardised mortality rates at the LTLA level, from regional data, is described in the Supplementary Appendix*") we agree that some elements could be referenced specifically and more frequently.

   On the inequality measures we add: Further detail on their creation is provided in the Supplementary Appendix.
   On the factor analysis we add: Additional information on the factor analysis undertaken is provided in the Supplementary Appendix

   Additionally, we move the entirety of the 'statistical analysis' section from the appendix and integrate it into the existing analysis section in the main text.

9. "Figure 1 – Line graph showing weekly relative inequality on the left hand y axis, and weekly average proportion of the whole population of each LTLA in quintiles one (most) and five (least) vaccinated by dose." Please consider also explaining the right-hand vertical axis in the figure caption of Figure 1.

   We now include the following:

   …the average proportion of the population vaccinated over time by dose.

10. "There were different patterns seen within each of the regions with higher inequalities seen in the midlands and northern regions, than seen in London and the South." Please consider describing these patterns in more detail with reference to the respective figures.

    We have added some detail, however we would caution over interpretation of the regional figures as some areas have small populations in the extremes of deprivation (relatively few in the most deprived or least deprived). In response, we add more detail on these patterns, referring back to regional graphs in the appendix:

Notably, inequality in cumulative mortality was very low in the South West and particularly high in the North East & Yorkshire, and the North West and East and West Midlands (Supplementary Figs 3d, 8a, 6a). Graphs overlaying the regional data (particularly Supplementary Figs 8a-10b) demonstrate particularly well the especially low inequality in the South West compared to other regions. However, absolute inequality in weekly mortality is highest in two large peaks in London, at the start of the pandemic and early in 2021 (Supplementary Figs 3a, 4a, 7a, 9a).

11. "The impact of the different uptake of the vaccinations is shown in Figure 2 and Table 1. Absolute inequality first plateaued then continued to increase but at a slower rate after the introduction of vaccinations, with relative inequality stabilising." For clarity, I suggest describing the results with "inequality in mortality 28 after a positive COVID-19 test" or "COVID-19 related mortality" instead of simply referring to inequality. Since inequalities in vaccination up-take is also of interest in this study, such clarity might avoid confusing the two inequalities.

    We are grateful to Reviewer 2 for highlighting this area of possible confusion and amend description of inequalities in this sentence as follows:

    Absolute disparity inequality in mortality 28 days after a positive COVID-19 test first plateaued then continued to increase but at a slower rate after the introduction of vaccinations, with relative inequality stabilising.

12. Lastly, there are many figures in the supplementary appendix which are not mentioned in the main text. Even if the authors lack the space to describe all their results, it may be worth linking these additional figures to the main text and justify their presentation.

    There are many additional graphs included in the appendix due to the number of inequality measures used and to demonstrate each region separately. We did not wish to over interpret the results and hope that our response to query 10 addresses this query too.

Reviewer 3

1. The first paragraph of the methods belongs in the introduction

   We thank Reviewer 3 for this suggestion and have moved this paragraph to the end of the introduction.

2. The abstract says that the study is of adults but no information on this is included in the methods

   We agree that this is unclear and have removed reference to the adult population here.

3. Given that this is an ecological study, the two primary measures need to be described as population measures, e.g. rather than deaths within 28 days of a positive test, it needs to be "age-standardised 28-day COVID-related mortality rates". The other outcome, "vaccination doses" isn't clear at all. Proportion of the population who received 1, 2 and 3 doses?

   Thank you for flagging this lack of clarity and consistency. We refer to the death data in this way initially as this is the data which we use to go on to calculate our age-standardised rate from. However, we agree that after this initial description of the data, the data we estimate should consistently be referred to as 'age-standardised mortality rates 28 days after a positive COVID-19 test'. We therefore go through the full manuscript and appendix and revise the phrasing of this where necessary.

   In relation to the vaccination data we change the phrasing of the variable resulting from the factor analysis to 'population vaccination load' where we refer to analyses including the vaccination factor which captures vaccinations one, two and three.

4. Their measure of vaccination uptake is not clear at all. The statement on page 7 line 57 "produced a cumulative proportion vaccinated measure" seems to suggest that the main measure is the proportion having received 1, 2, and 3 doses at 26 December 2022? And how this relates to LTLAs I'm not sure? Other places refer to average proportion in the quintile?

   The variable used in the analysis takes the weekly proportion of the LTLA population who have had 1, 2 and 3 doses over time and combines them to create one variable that indicates the vaccine level within that area. The vaccine level variable increases over time. These individual LTLAs are then used to investigate the effect of deprivation quintile. A region has multiple LTLAs and can have multiple areas within the same deprivation quintile. The quintile is used for the inequality measure.

5. It's unclear to me what went into the factor analysis – or why this was done – and what this measured was used for. Could the authors please clarify?

   We thank reviewer 3 for raising this issue which has arisen from a lack of clarity in the paper. Determining the efficacy of vaccination in a given area is complicated by the three dose regime recommended for most people in the UK. Factor analysis identifies underlying relationships between measured variables by reducing the number of observed variables into fewer unobserved variables. We estimated an unobserved variable for population

immunity using the observed vaccination rates for the first, second and third covid doses. In order to improve clarity, we have changed ‘overall estimate of vaccination’ to ‘population vaccine load’. In the appendix we have also added the following:

It is important to undertake an analysis that takes into account that vaccinations were cumulative, and that more immunity was incurred (and sustained) with additional vaccinations. Vaccinations were rolled out over a period of time however and as Figure 1 shows, this caused sudden increases in the proportion of the population with one, two or three vaccines. The variable measured as proportion of the population was also then influenced particularly by the third vaccination wave as the addition of the third vaccine meant that the proportion with just two vaccines then decreased. To analyse these each separately would have caused difficulties as before the introduction of the third vaccine, two vaccine doses was considered optimal, whereas after the roll out of the third dose, having only two doses was considered suboptimal.

6. The deprivation index includes a measure of health. What implications does this have for the findings? This needs to be incorporated into the discussion in some capacity because by my relatively quick thinking, this would underestimate inequalities in vaccine uptake between deprivation areas (i.e. more deprived areas have greater proportion of people in poor health, who had higher uptake of vaccines) but it might overestimate inequalities in mortality (more deprived areas have greater proportion of sicker people, who are at greater risk of mortality once infected).

   Reviewer 3 is correct to point out that the Index of Multiple Deprivation does include a health domain. The English IMD is designed to comprehensively capture relative area deprivation and health is just one of seven domains and contributes only 13.5% of the final area deprivation score. Health related studies excluding this domain typically find little change in their overall findings. One study in particular has specifically addressed this and found removing the health domain had little effect on analyses of socio-economic inequalities in health (Adams, J. and White, M., 2006. Removing the health domain from the Index of Multiple Deprivation 2004—effect on measured inequalities in census measure of health. Journal of Public Health, 28(4), pp.379-383.). Furthermore, as we use LTLAs which are a large geography and transform their deprivation ranks into quintiles, we therefore believe it is unlikely that removing the health domain would substantially alter the individual LTLA rank or the quintile it is ultimately allocated to.

7. I couldn’t follow the methods used to estimate age-standardised mortality rates in the supplementary material (more comments below). Additionally, given that this is the primary outcome, at least some of the detail on how this was estimated needs to go into the main text.

   We have rewritten the formulas included in the appendix in order to make the process followed to calculate age-standardised mortality easier to understand. In addition, to address the lack of clarity on the process undertaken in the main text, we add:

   Age-standardised deaths are not produced at the LTLA due to identification risks. We therefore undertook a multi-step process in order to calculate age-standardised deaths from data on regional age-stratified deaths, LTLA total deaths and LTLA population age structure.

8. The authors need to add their methods for estimating the counterfactual scenario of what inequalities in mortality rates would have been had vaccination uptake been the same across areas. How do they define "the same". In the supplementary file, it says if most deprived quintile had uptake the same as in least deprived quintile, but this needs to be in the main text.

   The counterfactual takes the within region average population vaccine coverage in the least deprived and uses that estimate and the relationship between population vaccine coverage and mortality estimated in the region to show what would have happened in the most deprived areas had vaccine coverage been better. It takes into account the differences in the region.

9. Given that this is aggregated, whole-of-population data, how do the authors interpret the confidence intervals?
   The confidence intervals mainly indicate that the estimates of inequality are produced within region and there are only nine regions in the analysis.

10. How is a region different to a LTLA? As someone who isn't familiar with the English data, I was confused by this.

    Thank you for highlighting this lack of detail. In addition to providing more information describing LTLAs:

    The size of LTLA populations ranges from 41,000 (Rutland) to 1,158,000 (Birmingham) with a mean of 194,000 persons (SD 130,000, SE 7,600) and a median of 149,000 persons. LTLAs are subcomponents of regions.

    We also add:

    regional (a larger geography than LTLAs) and There are nine regions in England which LTLAs are nested within.

11. How was age operationalised for these analyses? Single year of age or 10-year age groups? Or something else?

    We have added the following statements in brackets to clarify:

    We therefore undertook a multi-step process in order to calculate age-standardised deaths from data on regional (a larger geography than LTLAs) age-stratified deaths (by five year age bands), LTLA total deaths and LTLA population age structure (by five year age bands).

12. Throughout the text, can the authors clarify whether they are estimating death rates or death counts?

We first estimate death counts by age group as this is not available to us at the LTLA level. Following this, as part of the process for calculating age-standardised mortality, we calculate death rates. We hope that having now rewritten the formulas for this process in the appendix has improved the clarity of this. The analyses are all undertaken on death rates.

13. Can the authors clarify throughout the text if they are referring to all-cause or COVID-related deaths?

    The only death measure used in this paper is that of all deaths 28 days after a positive COVID-19 test which we take as a proxy for death with COVID-19 on the death certificate. The measure is therefore neither all-cause mortality nor, strictly speaking, COVID-related deaths. It was a common measure of mortality reported by the UK government during the COVID-19 pandemic. We appreciate that this may have been unclear in parts of the paper prior to the methods section where our death measure is formally described. We therefore add '28 days after a positive COVID-19 test' anywhere where the word 'deaths' was mentioned in relation to our analysis without this information, paying particular attention to the sentences in the methods section which we suspect may have contributed to the lack of clarity.

14. Page 19 line 24, is "data" a typo and should it say "date"? I struggled with that first sentence.

    Thanks for picking up on this, this has now been corrected to 'date'.

15. Page 19 line 36, what does the "(A) refer to?

    Thanks for picking up on this, this is a typo and has been removed.

16. Page 19, line 41, "to calculate the age-specific unknown numbers…" of what? Number of COVID-19 related deaths?

    To improve clarity, we add:

    of deaths 28 days following a positive COVID-19 test

17. The equations are not intuitive and seem to use "age" a bit inconsistently. Furthermore, unless I really misunderstood, the use of subscripts for LTLA and region also used quite inconsistently

    We have rewritten these and hope that they are now clearer?

18. There is only one heading "Step 1", which implies other steps, but no other headings for different steps?

    Within the text of the appendix there is a sentence that says the calculations "return to step 1" as it is an iterative procedure. It could say "beginning of loop", but we felt "step 1" more appropriate. By this we mean that there is no need for more steps, but that step 1 may occur several times.

19. I'm not sure that I follow, but if I understood correctly, the method used to get age-specific death rates for each LTLA imply that each LTLA has the same mortality as the "region" – is this interpretation correct? What evidence is there that this assumption is true? Or will it all average out in the end given that LTLAs make up the region?

    No the analysis does not have this assumption. The counts of deaths are given at both the LTLA and region level, with age-specific counts also available at the region level. For both the LTLA and region the age distribution of the population is known. The method therefore uses an iterative process to turn the measured age-specific data from the region into the constituent LTLAs so as to be consistent with the total numbers for both the LTLA and the region and the age profile of each LTLA. The assumption is more that after allowing for differences in the overall LTLA rate (which are measured) the age distribution of the deaths is not different between the LTLAs and is consistent with the age distribution of the deaths at the region level, after adjusting for differences in the age distribution of the population. We feel that this assumption is likely to be true.

    We hope that the re-writing of the formula for calculating this in the appendix, plus the additional detail on the difference between the geographies we have now added helps to make this clearer.

20. The authors talk about the estimates being adjusted for "over/ under estimation" (page 20, line 5), what does this mean? How was this done?

    We have rewritten the formulas and hope this is now clearer. Essentially when the first estimate is created for each LTLA by just imposing the region death rates an LTLA it could be over or under estimated compared to the measured death counts in the data from that LTLA. This difference is calculated and then used to adjust the estimates so that they are consistent with the observed data (at both LTLA and region level).

21. It isn't clear to me why the factor analysis was done or what went into the analysis? What does "100% proportion" refer to?

    Please see response to query number 5. Furthermore, '100% proportion' refers to the proportion of the total variance in vaccination explained by the factor variable.

    In the appendix we add: …with the first factor expressing 100% proportion of the variance.

22. Page 21, line 34 "inequality rate is defined as the quintiles of inequality converted to a 0/1 variable with the quintiles equally distributed". I'm not sure what this sentence means – could the author clarify? A dummy variable for each quintile? Equally distributed based on what, population within each LTLA area or equal number of LTLAs within each quintile?

    Quintiles are usually numbered 1 to 5 in the data to express least to most deprived. Using 1 to 5 is not efficient for analyses so values of between 0/1 are used for the quintiles, this means that the estimate in the models then reflects the change from most to least deprived. Quintiles are calculated at the population level. Each LTLA is in a single deprivation quintile,

but a region will have a number of LTLAs all at different quintiles. The quintiles are equally distributed. The recoding of the variable does not change the analysis but just changes the scale to enable direct estimation of the deprivation effect from low to high.

23. Why adjust for region? How do you interpret the results when adjusted for region?

We are not adjusting for region. The analysis is undertaken within the region. The difference between the most deprived and least deprived for each region has been used. This is to enable regional differences to be appropriately modelled.

24. I'm confused as to what their measures of inequality were. They seem to imply a difference between absolute and relative measures and the diversity index and the concentration index, but the diversity index looks like a relative measure to me (it looks like relative rate). I'm not sure what they modelled when they talk about relative and absolute measures?

We understand that confusion can be created by the different methods of estimating absolute and relative inequalities. The main difference between the different measures is whether the estimates are measured in absolute or relative terms, using the extremes (most versus least deprived) or a linear effect across all quintiles, and whether the estimates are just observed or modelled. We have carefully gone through the manuscript and changed the terminology to match the following. We have added this table to the Supplementary appendix.

**Observed inequalities**	
Disparity index	A ratio variable calculated by dividing the mortality rate estimates for the most deprived quintile by the least deprived quintile.
Absolute disparity inequality	The difference between mortality rate estimates of the most deprived quintile and least deprived quintile.
Concentration index	The concentration index captures the extent to which the mortality differs across LTLAs ranked by deprivation quintile. The concentration index is twice the area between the concentration curve and the 45° line indicating no relationship between the deprivation and mortality. It is a value which increases with greater concentration of mortality among the more deprived quintiles.
Generalised concentration index	An absolute inequality measure from the concentration index. Calculated by multiplying the concentration index by the mean mortality rate.
**Modelled inequalities**	
Absolute inequality	The estimated linear effect of deprivation on mortality rate across all five quintiles.
Relative inequality	The relative risk of the deprivation in the least to the most deprived estimated from the linear effect of deprivation as above.

25. On a related note, where they refer to absolute measures, the authors need to add the measure and the scale. What is the measure of the absolute inequality in Figure 2? Rate difference per 100,000?

The absolute measure we employ is the difference per 100,000 and now reflected in the text. We have added the following:

"absolute disparity measured by the difference in mortality estimates for the least to most deprived quintile" and well as adding "per 100,000" where we refer to absolute inequality in table and graph titles.

26. The statistical analysis section of the supplementary file needs to go into the main text, and needs to be edited for clarity. What was the outcome in these models and what where the exposures in the model? Why adjust for region? These models seem to be modelling the association between 28-day COVID mortality rates and lockdowns, which where not part of the original aims? Also, if lockdown applied universally (i.e. no variation in the measure between areas) what is this model achieving?

We agree and have now integrated this section of the appendix into the section of the main text with the same sub heading and have edited this for clarity. The outcome for the models is the mortality rate, the exposure is just lockdowns and population vaccine coverage. The lockdown models were utilised to show that the differences seen in absolute and relative inequality were not just a function of the lockdowns that happened during an overlapping time period as the vaccination campaign. It is quite possible that lockdowns impacted more deprived populations differently to least deprived (evidence that was seen in France https://pubmed.ncbi.nlm.nih.gov/33845798/). It was therefore important to understand the impact of vaccination coverage differences taking into account lockdowns. Region was used in the models to reflect differences in areas for differences in COVID-19 infection rates, different local lockdowns and other similarities within regions rather than between regions.

27. Can the authors add more detail on the LTLAs in England? Are they defined by geographical boundaries (i.e. England broken in 309 areas of roughly the same size) or population size? How many people on average live in these areas? This was in supplementary material but needs to go into the methods.

We appreciate reviewer three bringing this to our attention, this was also raised by reviewer two and we have added the following into the main text:

The size of LTLA populations ranges from 41,000 (Rutland) to 1,158,000 (Birmingham) with a mean of 194,000 persons (SD 130,000, SE 7,600) and a median of 149,000 persons.

28. The figures seem to have headings embedded in the images and in text on the page. Suggest just one.

    Thank you for highlighting this. We agree and remove the embedded titles.

29. In Figure 1, I would suggest reporting the absolute inequalities. Absolute differences are where inequalities play out in the real world. Plus, there are (what I assume to be) relative rates of ~50 in that figure. A RR of 50 is truly enormous and suggests a lot of uncertainty in the estimate. The underlying data are needed to interpret what's going on behind those big estimates.

    We thank reviewer three for raising this and switch Figure 1 in the paper with Figure 1 in the appendix so that the graph showing absolute inequalities is shown in the main text instead. We alter the text in paragraph two of the results section interpreting the graph to reflect this change.

30. The headings in Figure 2 are very non-descript and need to be updated. What are their measures of absolute and relative inequality?
    We have made clear the definitions of absolute and relative inequality in the text and have now provided further detail and a table for comparison in the appendix which we hope helps to remedy this lack of clarity. As authors we are not keen to repeat our methods in the headings of our figures. We have ensured that the Figure is referenced in the appropriate location and the methods have also now been made much clearer by removing duplicate terminology.

31. Table 1 is also quite unclear, including the heading. Absolute inequality measured in what scale? The CIs seem quite wide given the nature of the data, are they correct?

    The absolute inequality scale has now been added to the table. The confidence intervals reflect that the analysis relies on the relationship between the deprivation quintiles within a region and there are only nine regions. All regions have areas in the most deprived and least deprived quintiles, but to a varying extent. The evaluation of the model to reflect values that are consistent with the original scale of the data are expressed in the paper.

32. Page 9, line 37-38, the authors refer to "new mortality" which isn't a great way to describe what they are looking at (all deaths are new deaths). Can the authors revise?

    We agree that this is unclear and remove the word 'new' and replace it with 'weekly'.

33. Can the authors say what they found rather than describing what figures exist in the supplementary file?

    We now provide the following interpretation of these figures:

Notably, inequality in cumulative mortality was very low in the South West and particularly high in the North East & Yorkshire, and the North West and East and West Midlands (Supplementary Figs 3d, 6a, 8a). Graphs overlaying the regional data (particularly Supplementary Figs 8a-10b) demonstrate particularly well the especially low inequality in the South West compared to other regions. However, absolute inequality in weekly mortality is highest in two large peaks in London, at the start of the pandemic and early in 2021 (Supplementary Figs 3a, 4a, 7a, 9a).

34. The region-stratified analyses show quite a lot of variation in findings. E.g. the south west seems to buck the trend and have least disadvantaged areas having higher mortality rates relative to the more disadvantaged areas – can the authors comment on that? Is there anything to learn from different approaches taken by the different regions?

We have now added some detail describing these trends in response to the above comment, however we caution over interpretation of the regional data as some areas have small populations in the extremes of deprivation (for example some have relatively few in the most deprived or least deprived).

35. The purple lines in figure 2a exaggerate the points where the data are sparse. It might be better to either not present this figure, or to leave the purple areas completely blank and say that the RRs can't be estimated. And again, there are some really enormous RRs on that figure, which makes me think that the underlying data are quite uncertain.
These lines do not reflect uncertainty, but clear differences between the least and most deprived. The purple lines reflect points where deaths rates are zero in one deprivation quintile, but not zero in the other. These points are presented at a reasonable distance from the measured inequalities and in a different colour to show they are not true estimates. They are presented as the true estimate is unknown, but definitely not negligible. The fact that there are many more purple lines above the graph (deaths in most deprived with none in the least deprived) versus below the graph we feel gives valuable information. To exclude them would mean that if there were many weeks with no deaths in the least deprived but some deaths in the most deprived this data would be omitted.

36. What happened to COVID-19 testing over the time period? Are there estimates of what proportion of cases tests were capturing over time? And importantly, did this differ by socioeconomic position? What are the implications for bias of missing positive cases, particularly if ascertainment differs by socioeconomic position?

We provide a more detailed description of the COVID-19 test data and how this changed over time in the appendix. Though lab-based polymerase chain reaction (PCR) COVID-19 tests were no longer available to be independently ordered online for free to the general public after April 1st 2022, we anticipate this having little impact on our outcome measure of deaths within 28-days of a positive COVID-19 test. These lab-based tests have been available to hospitals and other health care professionals throughout the pandemic at no cost to the patient (thanks to the National Health Service).

Further, the majority of all COVID-19 deaths occurred in hospitals (75% of all COVID-19 deaths in 2021), followed by care homes (17%) (sheet 4a -

https://www.ons.gov.uk/peoplepopulationandcommunity/birthsdeathsandmarriages/deaths/datasets/deathsduetocovid19registeredinenglandandwales2020).

As our outcome is death 28 days after a positive test, rather than COVID-19 cases – we focus on the accuracy of our measure as a proxy for mortality COVID-19. In the discussion we provide detail on this: "*research suggests that the proportion of deaths within 28-days of a positive COVID-19 test which have COVID-19 listed as a factor on the official death certificate declined over as the pandemic progressed into 2022*.". Therefore, evidence suggests that our outcome measure may slightly overestimate deaths with COVID-19 on the certificate. Due to the availability of these tests to all healthcare professionals throughout the pandemic, we do not anticipate any strong bias in case ascertainment by SES or geography, and therefore we are confident that our outcome measure does not systematically mis-capture deaths by area deprivation.

We appreciate that these important details were perhaps not clear enough in the appendix and not present in the main text. Therefore, we additionally include:

"Furthermore, it is important to note that the majority of deaths with COVID-19 on the death certificate happened in clinical or care settings and that the change to limiting availability of PCR tests only to clinical settings is unlikely to have dramatically changed our outcome measure or to have led to any differences by geography or socio-economic status." (appendix). A citation is also provided to the data mentioned above on death location.

Further clarity surrounding test type and availability detail is provided in the main text including "Further information on changes to access to COVID-19 testing for the general public and discussion surrounding the potential implications of this is also provided in the Appendix."

37. The authors note that they have conducted an ecological study but don't really address what specific limitations this type of study design has? I'm concerned about this, particularly given that health is included in the definition of deprivation.

    We agree with reviewer three that caution is needed when interpreting the results of studies using ecological data. In acknowledgement of this, we had included the following in the paper "*However, several limitations should be considered. Firstly, it is important to note that this is an ecological study. The data reflect the qualities of LTLAs and not the individuals within them*". We now further clarify:

    Therefore, our conclusions surrounding vaccination and mortality inequality pertain specifically to population effects rather than an individual effect.

    Further, we address concerns surrounding the inclusion of the health domain in the Index of Multiple Deprivation in comment number 6.

38. The authors have had to estimate age-standardised mortality at the LTLA level, which is the primary outcome, but don't discuss what assumptions they've made in doing so, or the implications for bias this creates.

We check our LTLA age-stratified estimates against the known total number of deaths 28 days after a positive COVID-19 test, ensuring our estimates do not exceed these numbers. This means that there should be no bias by area deprivation quintile in these estimates. However, is theoretically possible that the age distribution of mortality 28 days after a positive COVID 19 test differs by area deprivation. If this were true, it would mean that the age standardisation may not be accurate. However, the evidence overwhelmingly suggests that the difference between less and more deprived areas is in the number of deaths, rather than their distribution across age groups. We add the following to make this assumption clearer:

In estimating the mortality data in this way, we assume a uniform distribution of mortality rates by age across differing levels of area deprivation within each region.

And
Relatedly, we had to estimate LTLA age-adjusted deaths from regional age-stratified deaths. Though we perform tests to ensure these estimates are consistent with the known total number of deaths 28 days after a positive COVID-19 test for each LTLA, it is possible that the age distribution of deaths in each LTLA could differ from what we assume after taking account of the population structure.

39. We can't determine "the effect" of something on something else with an ecological study. The authors need to revise the manuscript, including the title, to remove causal language.

    In response, we change 'impact' and 'effect' where used in reference to our analyses to 'association' throughout the manuscript.

40. The future directions and conclusions are not particularly related to the findings of the study, could they be revised.

    We respectfully disagree with reviewer 3 on this point. The conclusions made are directly informed by the findings of the study. We make suggestions as to the possible focus of future research which build on the findings of our study only in the very last sentence of our article and feel that this is appropriate at this point in the paper.

41. There are a few typos and points where important commas are missing. Could the authors revise for clarity.

    We have corrected all typos brought to our attention. We have carefully read this updated manuscript draft and supplementary material and have added in any commas in where we felt they may help improve readability.

VERSION 2 - REVIEW

**Reviewer**	**2**
**Name**	**Oberndorfer, Moritz**
**Affiliation**	**University of Helsinki**

Date **26-Nov-2024**

COI

The authors have provided sufficient clarity and detail so that readers can understand and evaluate the study themselves. The authors have addressed my comments sufficiently.

Respecting open science principles, I would still suggest publishing the code for the statistical analyses shown in this paper and provide a link to it in the paper. In my view, having done the analysis in Stata is not an obstacle to publishing the code as indicated by the authors' response. Even if readers do not have access to Stata to run the code, they can still read the code.

VERSION 2 - AUTHOR RESPONSE

Reviewer: 2
Comments to the Author:
The authors have provided sufficient clarity and detail so that readers can understand and evaluate the study themselves. The authors have addressed my comments sufficiently.
Respecting open science principles, I would still suggest publishing the code for the statistical analyses shown in this paper and provide a link to it in the paper. In my view, having done the analysis in Stata is not an obstacle to publishing the code as indicated by the authors' response. Even if readers do not have access to Stata to run the code, they can still read the code.

We agree in principle code should be shared wherever possible. Unfortunately in this case, two difficulties exist. The code for this work forms part of a much larger project and therefore would require significant editing to ensure just this analysis is run. Secondly, in this case, the length of time taken in the publication means that many of the team have moved on and code has been archived. Our suggestion instead is that it can be provided upon request. This would enable a request to the host institution to unarchive the research space. We include the following statement in the appendix to flag this:

"The code used for the analyses in this paper is available from the authors upon request."
